# Computerized Analysis of Verbal Fluency: Normative Data and the Effects of Repeated Testing, Simulated Malingering, and Traumatic Brain Injury

**DOI:** 10.1371/journal.pone.0166439

**Published:** 2016-12-09

**Authors:** David L. Woods, John M. Wyma, Timothy J. Herron, E. William Yund

**Affiliations:** 1 Human Cognitive Neurophysiology Laboratory, VANCHCS, Martinez, CA, United States of America; 2 UC Davis Department of Neurology, Sacramento, CA. United States of America; 3 Center for Neurosciences, UC Davis, Davis, CA United States of America; 4 UC Davis Center for Mind and Brain, Davis, CA United States of America; 5 NeuroBehavioral Systems, Inc., Berkeley, CA United States of America; University of California, San Francisco, UNITED STATES

## Abstract

In verbal fluency (VF) tests, subjects articulate words in a specified category during a short test period (typically 60 s). Verbal fluency tests are widely used to study language development and to evaluate memory retrieval in neuropsychiatric disorders. Performance is usually measured as the total number of correct words retrieved. Here, we describe the properties of a computerized VF (C-VF) test that tallies correct words and repetitions while providing additional lexical measures of word frequency, syllable count, and typicality. In addition, the C-VF permits (1) the analysis of the rate of responding over time, and (2) the analysis of the semantic relationships between words using a new method, Explicit Semantic Analysis (ESA), as well as the established semantic clustering and switching measures developed by Troyer et al. (1997). In Experiment 1, we gathered normative data from 180 subjects ranging in age from 18 to 82 years in semantic (“animals”) and phonemic (letter “F”) conditions. The number of words retrieved in 90 s correlated with education and daily hours of computer-use. The rate of word production declined sharply over time during both tests. In semantic conditions, correct-word scores correlated strongly with the number of ESA and Troyer-defined semantic switches as well as with an ESA-defined semantic organization index (SOI). In phonemic conditions, ESA revealed significant semantic influences in the sequence of words retrieved. In Experiment 2, we examined the test-retest reliability of different measures across three weekly tests in 40 young subjects. Different categories were used for each semantic (“animals”, “parts of the body”, and “foods”) and phonemic (letters “F”, “A”, and “S”) condition. After regressing out the influences of education and computer-use, we found that correct-word z-scores in the first session did not differ from those of the subjects in Experiment 1. Word production was uniformly greater in semantic than phonemic conditions. Intraclass correlation coefficients (ICCs) of correct-word z-scores were higher for phonemic (0.91) than semantic (0.77) tests. In semantic conditions, good reliability was also seen for the SOI (ICC = 0.68) and ESA-defined switches in semantic categories (ICC = 0.62). In Experiment 3, we examined the performance of subjects from Experiment 2 when instructed to malinger: 38% showed abnormal (p< 0.05) performance in semantic conditions. Simulated malingerers with abnormal scores could be distinguished with 80% sensitivity and 89% specificity from subjects with abnormal scores in Experiment 1 using lexical, temporal, and semantic measures. In Experiment 4, we tested patients with mild and severe traumatic brain injury (mTBI and sTBI). Patients with mTBI performed within the normal range, while patients with sTBI showed significant impairments in correct-word z-scores and category shifts. The lexical, temporal, and semantic measures of the C-VF provide an automated and comprehensive description of verbal fluency performance.

## Introduction

In verbal fluency (VF) tests such as the Controlled Oral Word Association Test (COWAT)[1], subjects retrieve as many words as possible in semantic (e.g., “animals”, “foods”, etc.) or phonemic (e.g., words beginning with “F”, “A”, or “S”) categories during a limited period of time (usually 60 s). Verbal fluency tests are routinely used to evaluate cognitive function in clinical disorders, including Alzheimer’s disease [2], Huntington’s disease [3], attention deficit disorders [4], traumatic brain injury (TBI) [5], and aphasia [6, 7]. Correct-word scores reflect lexical retrieval [8, 9] and executive control [10], and are most severely impaired following lesions of the left frontal and left temporal lobes [11, 12].

Oral VF tests were introduced by Benton and colleagues in the 1960’s [13] and are still routinely used to evaluate memory retrieval in neuropsychiatric and developmental disorders [14–16]. Test administration and scoring procedures have remained largely unchanged over the six decades since VF testing was introduced: investigators typically transcribe the words with pencil-and-paper and tally the total number of correct words retrieved (i.e., total words minus repeated words and out-of-category words). Here, we describe a computerized VF test (C-VF) that standardizes test administration and scoring and permits the automated analysis of lexical, temporal, and semantic factors that provide further insight into VF performance.

Table 1 summarizes the mean correct-word scores of recent large-scale VF studies. Despite the apparent simplicity of the test, there are significant discrepancies in the correct-word scores obtained in different normative groups of similar age and education. For example, the 40–49 year old subjects in the Delis-Kaplan Executive Function System (D-KEFS) normative data set [17] retrieved 18.33 correct words in the “animals” semantic category. This was 0.20 standard deviations below the age-matched norms (20.7) of Tombaugh et al. (1999) [18] [t(94) = -1.08, NS], 0.73 standard deviations below the age-matched Caucasian norms (23.0) of Gladsjo et al. (1999) [19] [t(187) = -4.54, p <0.0001], and more than one standard deviation below the German “animal” norms (26.2) of Then et al. (2014) [20] [t(116) = -10.40, p < 0.0001].

**Table 1:** Large scale studies of verbal fluency since 1999.

STUDY	CHACTERISTICS			SEMANTIC			PHONEMIC			
	No.	Age	Edu	CW	SD	CV	CWs	SD	CV	"F"
**Gladsjo et al, 1999**	768	52.12	14.09	19.54	5.39	0.28	40.34	12.6	0.31	14.07
**Sugarman et al., 2015**	431	49.60	13.10	18.40	5.30	0.29	33.60	11.90	0.35	11.72
**Hankee et al, 2013**	1907	67.00	15.00	18.10	5.10	0.28	38.00	12.4	0.33	13.25
**de Azeredo Passos et al, 2015**	4900	55.00	14+	20.00						14.00
**Vanderploeg et al., 2005**	3057	38.42	13.27	20.49	5.09	0.25	34.63	10.75	0.31	12.07
**van Der Elst et al, 2006**	1825	51.62	11.22	19.80						
**Then et al, 2014**	2927	62.00	12.00	23.50	6.22	0.26				
**Hoogendam et al, 2014**	4422	71.80	12.50	21.50	5.80	0.27				
**Feeney et al, 2013**	4553	62.80	12.00	20.50	9.60	0.47				
**van Hooren et al, 2009**	574	71.00	12.00	20.90	5.50	0.26				
**Wecker et al., 2005**	719	50.96	13.11	18.33	4.64	0.25				
**Tombaugh et al. 1999**	735	67 .40	11.4	16.90	5.00	0.30				
**Ammann et al, 2013**	2157	73.00	14.00				39.70	12.50	0.31	13.84
**Tombaugh et al. 1999**	1300	52.30	12.90				37.50	13.10	0.35	14.40
**Zhong et al., 2014**	952	73.30	12.90				33.80	11.80	0.35	11.78
**Mean**	**2082**	**59.89**	**12.87**	**19.83**	**5.76**	**0.29**	**36.80**	**12.14**	**0.33**	**13.14**
**Experiment 1**	180	40.03	14.52	20.09	4.88	0.24	14.64	4.96	0.34	14.64

Edu = approximate years of education; CW = correct words; SD = standard deviation; CV = coefficient of variation; “F” = estimated number of words beginning with “F” in phonemic conditions (F-words were 34.9% of FAS word totals in Tombaugh et al., 1999). Semantic conditions were “animals”. Data from Experiment 1 show values after 60 s for “animals” and letter “F”. The de Azeredo Passos results show medians (not means) for highly educated middle-aged subgroup of Brazilian civil servants.

These discrepancies likely reflect differences in test administration and scoring [21], language effects [22], and differences in culture [19]. Test administration procedures may differ as to when the 60 s test begins (e.g., with the first word articulated or with the “begin” command), and vary in the extent to which words articulated at the end of the test period are included in the correct-word score. There may also be differences in procedures for correcting errors, classifying ambiguous responses (e.g., “dinosaur” in the animal category), and encouraging subjects to continue producing words late in the test period.

Scoring procedures can also differ. For example, some examiners exclude subcategory names (e.g., “fish”) from correct-word scores when members in the subcategory (e.g., “trout”) are retrieved [23], while others include both words. Moreover, although inter-rater scoring reliability is generally high [24], correct and repeated words are tallied manually, introducing possible scoring errors.

On average, about seven words are retrieved in the first 15 s of the semantic fluency test [25, 26], a production rate (i.e., 28 words-per-minute) that exceeds typical handwriting speed (14 to 18 words-per minute) [27]. As a result, response transcription often falls behind report. Transcription complexity also varies with test format. For example, in the D-KEFS version of the VF test, words are transcribed onto different portions of the scoring sheet during each 15 s interval, so that the examiner will sometimes be transcribing one word, listening to another, and, at the same time, monitoring elapsed time and deciding where to write the next response.

While it is easy to tally correct-word scores, the analysis of the lexical, temporal, and semantic characteristics of word retrieval is more challenging and is rarely performed outside of research laboratories. However, previous studies show that these supplementary measures enhance the clinical sensitivity of VF testing, as described below.

### Lexical measures of verbal fluency

Several lexical measures have proven useful in interpreting VF test results. For example, studies have found that subjects who use frequent, typical words have low correct-word scores [28]. Juhasz et al. (2014) [28] compared the performance of patients with schizophrenia and controls and found that schizophrenics retrieved more frequent, typical words. Vita et al. (2014) [29] studied patients with mild cognitive impairment (MCI) and Alzheimer’s disease (AD). Both the MCI and AD groups used more typical words than controls. Moreover, the typicality scores in MCI patients were more predictive of their conversion to AD than their correct-word scores.

### The temporal decline of word production

The rate of word retrieval declines sharply over the retrieval period [30–33] with subjects typically retrieving roughly two-thirds of their word total during the first half of the test [25]. Fernaeus and colleagues (1998) [34] argued that retrieval in the early and late portions of the test reflected semi-automatic and effortful processes, respectively, and found that patients with AD [35] and white-matter hyperintensities [36] showed disproportionate reductions early in the test. Others have found early-retrieval deficits in patients with traumatic brain injury [37] and children with attention deficit hyperactivity disorder (ADHD) [38].

### Semantic analysis of verbal fluency

In VF testing, words are generally retrieved in semantically related clusters [26, 32, 33]. Troyer et al. (1997) [39] developed a widely used procedure for analyzing semantic clusters in the “animals” category. They defined 22 subcategories of animals based on living environment (e.g., Africa, North America, Australia, etc.), human use (e.g., pets, beasts of burden, animals used for their fur), and zoological classification (e.g., felines, canids, primates, etc.). They found that young subjects retrieved 21.8 words during the 60 s test, with 10.6 switches between subcategories, whereas older subjects retrieved fewer words and showed a corresponding reduction in the number of subcategory switches. In a subsequent study [40], they found that patients with lesions of the left frontal lobe showed a reduction in the number of switches between subcategories, while patients with lesions of the left temporal lobe showed a reduction in the size of clusters. Subsequent studies have used the subcategory classification methods to study semantic organization during language development [41], aging [42, 43], and in clinical populations with Alzheimer’s disease [44], schizophrenia [45], and TBI [46, 47].

Despite this fruitful line of research, there are several limitations associated with the use of *a priori* subcategories. First, subcategories must be defined for each category tested (e.g., “animals”, “cars”, “foods”, etc.). In addition, many words can be assigned to multiple subcategories. For example, in the classification scheme of Troyer et al. (1997) [39], a rabbit is classified as a North American animal, a pet, a farm animal, and an animal used for its fur. This results in ambiguity in identifying the words associated with subcategory switches. For example, there are no clear subcategory switches in the Troyer-based analysis in the seven-word sequence “rabbit, cat, tiger, lion, zebra, crocodile, whale” because words 1 and 2 are pets, words 2, 3, and 4 are felines, words 4, 5, and 6 are African animals (tigers were incorrectly categorized as African animals), and words 6 and 7 are water animals. Thus, while there are four subcategories, at no point is a word associated with an unambiguous switch between subcategories because “cat” is both a pet and a feline, “lion” is both a feline and an African animal, and “crocodile” is both an African animal and a water animal.

Although different subcategorization schemes have been proposed by different authors [48–51], any *a priori* subcategorization scheme necessarily represents only a small fraction of possible subcategories. For example, in the Troyer et al. (1997) [39] scheme, there is no separate subcategory for “Ocean” animals: whales, Orcas, and sea lions are included with frogs, toads, and alligators in the “water animal” subcategory. North American, Arctic, African, and Australian animals are defined subcategories, but there are no subcategories for South American, Asian, or European animals, nor are there subcategories for animals commonly hunted (e.g., rabbits, ducks, deer, etc.), or fish commonly taken for sport (e.g., trout, salmon, etc.). In addition, guidelines are lacking for categorizing supra-ordinate responses (e.g., “mammal”, “quadruped”), extinct animals (e.g., “dinosaur”, “T-Rex”), and imaginary animals (e.g., “unicorn”, “Big Foot”). Finally, the manual classification of words into subcategories is time-consuming, shows only moderate test-retest reliability [52], and can result in discrepant scores from different raters [53].

Several investigators have therefore turned to computational tools for measuring the strength of semantic associations between words. Ledoux et al. (2014) [48] used latent semantic analysis (LSA) [54], which reflects the co-occurrence of words in large text corpora, to quantify the semantic relationships between successive words. They found that LSA measures of semantic association were stronger for words that fell within predefined Troyer-like subcategories than for switches across subcategories. Hills et al. (2012) [55] analyzed VF performance in a 3-minute test using the Troyer method and a computerized semantic analysis method that combined LSA-type analysis with information about word order [56]. Although semantic association strengths varied substantially within the Troyer-defined clusters, they were markedly reduced when successive words switched between Troyer subcategories.

In the current manuscript, we analyzed semantic relationships using the Troyer classification scheme and a new computational method, Explicit Semantic Analysis (ESA) [57]. Explicit Semantic Analysis quantifies the relationships between words in a “concept space” defined from an analysis of Wikipedia entries [57]. Unlike *a priori* subcategory methods, ESA quantifies the strength of semantic associations on a continuously varying scale based on the strength of the association of word concept vectors derived from the analysis of Wikipedia Entries. This enables ESA to analyzed phrases like “Bernanke takes charge” to determine that it refers to Ben Bernanke and connects conceptual categories including the Federal Reserve Bank, the Chairman of the Federal Reserve Bank, Monetarism, and Inflation and Deflation [57]. Such analyses are difficult for LSA-like methods that depend on the co-occurrence of words in text.

Explicit Semantic Analysis measures the semantic relationship between words as cosine measures of their concept vectors [58]. Thus, ESA captures the semantic relatedness of words based on an exhaustive analysis of all possible conceptual similarities (e.g., taxonomic, geographic, economic, linguistic, cultural, utilitarian, etc.). As a result, the association strength between successive words (the pairwise ESA or PW-ESA) can differ markedly from those obtained with *a priori* subcategory classification schemes. For example, the words “tiger” and “shark” fall into separate, pre-defined Troyer subcategories (African animals and water animals). However, “tiger” and “shark” have strong associations in ESA concept space (e.g., both are threatening apex predators) and the two words occur together in the species name “tiger shark”. Thus, the PW-ESA cosine measure of the association between “tiger” and “shark” exceeds that of many word pairs (e.g., “ostrich and “monkey”) that are included in the same Troyer subcategory (i.e., African animals). Conversely, “toad” and “whale” show low PW-ESA association strengths, but are included within the same Troyer subcategory (water animals).

We describe four C-VF experiments that analyze standard VF scores (correct words and repetitions), lexical measures (word frequency, length, and typicality), temporal decline in the rate of word retrieval, and the semantic organization of word retrieval using Troyer methods and novel ESA techniques. In Experiment 1, 180 subjects (ages 18 to 82 years) were studied to characterize the influence of demographic factors (e.g., age, education, and sex) on these performance metrics.

Relatively little is known about the psychometric properties of lexical, temporal, and semantic measures of VF performance. In Experiment 2, a group of 40 young subjects underwent three test sessions at weekly intervals. The first session (Experiment 2a) was used to evaluate whether the regression functions developed in Experiment 1 could account for the performance of subjects in Experiment 2. Experiment 2b and 2c were used to analyze the test-retest reliability of lexical, temporal, and semantic measures of VF performance.

Experiment 3 investigated the effects of simulated malingering on VF performance using the participants from Experiment 2. The goal was to determine whether simulated malingerers with abnormal correct-word scores could be discriminated from control subjects with abnormal correct-word scores based on the analysis of lexical, temporal, and semantic measures.

Finally, in Experiment 4, we investigated C-VF performance in 25 patients who had suffered mild or severe TBI. Previous studies have suggested that patients with mild TBI generally have correct-word scores within the normal range, while patients with severe TBI generally show deficits [5]. However, little is known about the effects of TBI on lexical, temporal, and semantic measures of VF performance.

## Experiment 1. Demographic Influences on Verbal Fluency

In Experiment 1, we studied 180 subjects ranging in age from 18 to 82 years to analyze the effects of age, education, and sex on correct-word scores in semantic (“animals”) and phonemic (“F”) conditions. Previous studies have generally shown significant age-related declines in correct-word scores [18, 59–62], with larger declines in semantic than phonemic conditions [18, 24, 63–65]. An age-related increase in the incidence of repeated words has also been reported [66].

Education is also strongly correlated with correct-word scores [20, 62, 64, 67]. Because education levels increased throughout the 20^th^ century, there has been an attendant increase in correct-word scores in cross-sectional samples tested at decade intervals [68]. As a result, correlations of age with correct-word scores in cross-sectional studies may overestimate the influence of age itself, unless education is also factored out [69].

Variable effects of sex on VF performance have been reported: many studies have failed to find significant sex differences [42, 60, 70], while others have found that women have superior performance [62, 69, 71]. Sex differences are further complicated by the different familiarity of men and women with particular semantic categories. For example, men typically retrieve more words than women when tested with “cars” and “tools”, while women retrieve more words than men when tested with “fruits” [23, 59, 72]. However, most previous studies have found no significant sex differences in the “animals” category used here [59, 70].

### Experiment 1: Methods

#### Ethics statement

Subjects in all experiments gave informed written consent following procedures approved by the Institutional Review Board of the Veterans Affairs Northern California Health Care System (VANCHCS) and were paid for their participation.

#### Subjects

We studied 180 control subjects, whose demographic characteristics are included in Table 2. The subjects ranged in age from 18 to 82 years (mean age = 40.0 years) and had an average education of 14.5 years. Sixty-one percent were male.

**Table 2:** Mean scores and standard deviations from all experiments.

DEMOGRAPHICS					SEMANTIC CONDITION														PHONEMIC CONDITION						
	No	Age	Edu	C-use	CW	RW%	TYP	SOI	LWF	SYLL	ESW	EMW	ECS	TDP	TSW	TMW	TCS	S-Z	CW	RW%	SOI	LWF	SYLL	TDP	P-Z
**E1**	**180**	**40.0***21*.*2*	**14.5***2*.*0*	**5.1***1*.*9*	**26.6***6*.*9*	**1.3***3*.*3*	**55.5***11*.*7*	**2.1***0*.*7*	**3.6***0*.*09*	**1.84***0*.*23*	**9.20***3*.*07*	**5.72***1*.*86*	**4.06***1*.*06*	**0.63***0*.*10*	**12.1***3*.*8*	**6.3***2*.*0*	**3.2***0*.*7*	**0.00***1*.*00*	**18.8***6*.*5*	**2.6***5*.*9*	**1.4***0*.*8*	**3.78***0*.*05*	**1.22***0*.*53*	**0.65***0*.*11*	**0.00***1*.*00*
**E2a**	**40**	**25.9***5*.*6*	**14.9***1*.*3*	**5.9***1*.*5*	**29.5***5*.*2*	**3.1***3*.*6*	**42.7***15*.*3*	**2.2***0*.*6*	**3.7***0*.*04*	**1.87***0*.*22*	**10.38***2*.*86*	**6.43***1*.*57*	**4.15***1*.*31*	**0.64***0*.*08*	**14.4***4*.*0*			**0.29***0*.*86*	**21.0***6*.*9*	**3.2***5*.*1*	**1.5***1*.*0*	**3.74***0*.*07*	**1.47***0*.*30*	**0.67***0*.*09*	**0.22***1*.*12*
**E2b**	**Body parts, “A”**				**33.6***6*.*6*	**1.4***2*.*0*		**2.4***0*.*6*	**3.7***0*.*06*	**1.59***0*.*22*	**9.28***2*.*51*	**6.55***1*.*68*	**4.82***1*.*26*	**0.64***0*.*10*					**19.6***6*.*4*	**2.0***3*.*2*	**1.8***1*.*4*	**3.72***0*.*18*	**2.38***0*.*36*	**0.63***0*.*09*	
**E2c**	**Foods, “S”**				**32.3***6*.*5*	**0.6***1*.*4*		**2.0***0*.*8*	**3.6***0*.*06*	**2.11***0*.*21*	**11.23***3*.*37*	**6.63***1*.*72*	**4.16***0*.*87*	**0.57***0*.*06*					**25.8***7*.*1*	**1.2***2*.*0*	**1.8***1*.*1*	**3.74***0*.*04*	**1.53***0*.*29*	**0.60***0*.*08*	
**E3**	**Malingering**				**20.1***5*.*8*	**9.1***10*.*7*	**60.2***22*.*5*	**1.9***0*.*8*	**3.7***0*.*07*	**1.70***0*.*33*	**7.50***3*.*39*	**4.35***1*.*72*	**3.18***1*.*70*	**0.64***0*.*07*	**11.0***4*.*2*			**-1.18***0*.*94*	**16.8***5*.*2*	**11.2***13*.*69*	**1.3***0*.*6*	**3.79***0*.*05*	**1.51***0*.*25*	**0.61***0*.*07*	**-0.46***0*.*87*
**E4 mTBI**	**21**	**34.3***11*.*2*	**13.5***1*.*4*	**5.14***1*.*88*	**27.1***6*.*4*	**0.7***1*.*4*	**39.9***17*.*9*	**2.3***0*.*6*	**3.7***0*.*05*	**1.86***0*.*30*	**8.71***2*.*57*	**5.62***1*.*56*	**4.19***0*.*88*	**0.59***0*.*11*	**11.1***3*.*3*			**0.20***1*.*13*	**18.2***6*.*6*	**3.1***4*.*74*	**1.1***0*.*4*	**3.79***0*.*04*	**1.46***0*.*23*	**0.65***0*.*10*	**-0.04***1*.*08*
**E4 sTBI**	**4**	**46.0***9*.*0*	**13.0***1*.*2*	**4.5***2*.*7*	**18.5***9*.*9*	**5.7***4*.*6*	**66.4***42*.*2*	**2.3***1*.*1*	**3.7***0*.*04*	**1.79***0*.*21*	**5.25***4*.*57*	**4.50***2*.*65*	**4.11***0*.*39*	**0.68***0*.*10*	**8.5***5*.*5*			**-1.01***1*.*22*	**13.3***6*.*7*	**2.8***5*.*6*	**0.8***0*.*3*	**3.76***0*.*09*	**1.76***0*.*37*	**0.71***0*.*14*	**-0.74***0*.*80*

Abbreviations: E1 = Experiment 1. C-use = hours of daily computer use. CW = correct words; RW% = percentage of repeated words; TYP = typicality, the median number of participants who produced each word (higher numbers indicate more typical words); SOI = semantic organization index; LWF = log word frequency; SYLL = mean syllable count; ESW = number of ESA-defined semantic switches; EMW = number of ESA multi-word clusters; ECS = average size of ESA multi-word clusters; TDP = temporal decline index, the percentage of words produced during the first half of the test. TSW = Troyer switches. TMW = Troyer multi-word clusters. TCS = Troyer multi-word cluster size. S-Z = z-score, corrected for age and computer use in semantic conditions. P-Z = z-score in phonemic conditions. In Experiment 2b, the semantic condition was “parts of the body” and the phonemic letter was “A”. In Experiment 2c, the semantic condition was “foods” and the phonemic letter was “S”. In all other Experiments, the semantic condition was “animals” and the phonemic letter was “F”. Tests lasted 90 s. Numbers in italics show the standard deviations for each cell.

Subjects were recruited from advertisements on Craigslist (sfbay.craigslist.org) and pre-existing control populations. They were required to meet the following inclusion criteria: (a) native English speaker; (b) no current or prior history of psychiatric illness; (c) no current substance abuse; (d) no concurrent history of neurologic disease known to affect cognitive functioning; (e) on a stable dosage of any required medication; (f) auditory functioning sufficient to understanding normal conversational speech; and (g) visual acuity normal or corrected to 20/40 or better. Subject ethnicities were 64% Caucasian, 12% African American, 14% Asian, 10% Hispanic/Latino, 2% Hawaiian/Pacific Islander, 2% American Indian/Alaskan Native, and 4% “other”. The population was somewhat unusual because of the high levels of education among older volunteers: 47% of the subjects older than 65 years had completed college, compared to 11.7% of adults over 65 in the 2009 US census.

#### Procedure

Verbal Fluency was the sixth test in the California Cognitive Assessment Battery (CCAB) and required 4–5 minutes per subject. Each CCAB test session included the following computerized tests and questionnaires: finger tapping [73, 74], simple reaction time [75, 76], Stroop, digit span forward and backward [77, 78], verbal list learning, visuospatial span [79, 80], trail making [81], vocabulary, design fluency [82], the Wechsler Test of Adult Reading (WTAR), choice reaction time [75, 83], risk and loss avoidance, delay discounting, the Paced Auditory Serial Addition Task (PASAT) [84], the Cognitive Failures Questionnaire (CFQ) and the Posttraumatic Stress Disorder Checklist (PCL) [85], and a local traumatic brain injury questionnaire. Testing was performed in a quiet room using a standard Personal Computer (PC) controlled by Presentation® software (Versions 13 and 14, NeuroBehavioral Systems, Berkeley CA).

Because many of the CCAB tests required subjects to respond with the mouse, we also recorded subject computer-use on a separate questionnaire using an 8-point Likert scale, with the options of “1: Never; 2: Less than 1 hour per week; 3: Less than 1 hour per day; 4: 1–2 hours per day; 5: 2–3 hours per day; 6: 3–4 hours per day; 7: 4–6 hours per day; 8: More than 6 hours per day”. Subjects reported an average computer-use score of 5.09 (an average of 2–3 hours per day). In previous studies, we found that daily hours of computer-use correlated with performance both on tests that required responding with the mouse [75, 76, 79, 81, 85] and tests that required only verbal output, such as digit span [78] and the paced auditory serial addition test [84].

#### Software

An executable, open-source version of the C-VF test is available for Windows computers at http://www.ebire.org/hcnlab/programs.htm along with a Python program that can score “animal” fluency test results to provide measures of word syllable count, word frequency, word typicality, and the number of repeated words, while also performing semantic analyses using both ESA and Troyer methods. Excel spreadsheets of the data are available at https://dx.doi.org/10.6084/m9.figshare.4220619

#### Apparatus and stimuli

Subjects were instructed to produce as many words as possible during two 90 s tests: (1) phonemic fluency (letter “F”) and (2) semantic fluency (“animals”), with the same test order used for all subjects. Before each test, subjects were told that proper nouns, repetitions, derivatives, and words outside the category would not be accepted.

The examiner, sitting to the left of the subject, typed each word or abbreviation as rapidly as possible. The use of the keyboard facilitated word transcription since typing speed (30–40 words-per-minute) [86] is typically about twice the speed of handwriting. In addition, the time of occurrence of the first letter in each word was logged and analyzed to examine the timecourse of word production.

After 90 s, the experimenter told the subject that the test was over. After the test, the experimenter edited the words for spelling errors and expanded words that had been abbreviated to permit lexical and semantic analysis.

#### Lexical analysis

The average frequency of each word was quantified from the American National Corpus database [87]. Word frequencies were transformed into log word-frequency. A syllable count was also obtained to quantify word length. In order to quantify word typicality, we created a list of animal names produced by the 220 control subjects in Experiment 1 and Experiment 2a and sorted the list by the number of subjects who produced each word. Words differed greatly in typicality. For example, more than 80% of subjects produced the words “cat” and “dog”, while more than 180 animal names were produced by only a single subject. Overall, the 30 most frequent animal names accounted for 50.1% of all words produced.

Because typicality scores were highly skewed (i.e., by words produced by only a few subjects), we quantified the median typicality of the words produced. Typicality scores were converted into percentages by dividing median typicality by the total number of subjects. Typicality scores ranged from 8.6% for the subject who produced the least typical words to 41.8% for the subject who produced the most typical words, with an average of 25.2% for the entire population.

#### Temporal analysis

The latency of the first letter typed by the experimenter was used to estimate the onset latency of each word and calculate interword intervals. In comparison with voice trigger measures of word onset latencies in seven subjects, first-letter typing latencies averaged 0.87 s (SD = 0.44 s), with 95% of latencies below 1.87 s. We found a very strong correlation between interword intervals measured using voice trigger and typing latencies [r = 0.984, t(200) = 76.88, p<0.0001]. Word latencies were used to assign words to six bins, each 15 s in width. The temporal decline percentage (TDP), the percentage of words retrieved during the first half of the test relative to total word production, was used to summarize the rate of temporal decline for each subject.

#### Troyer analysis of switches and clusters

Words gathered during semantic (“animals”) testing were assigned to 22 non-exclusive subcategories based on living environment (e.g., Africa, North America, Artic/Far North, etc.), human use (e.g., pets, farm animals, etc.), and taxonomy (e.g., primates, fish, etc.), following the procedures described in Troyer et al. (1997) [39]. Switches were defined as transitions between categories. The number of switches was obtained along with the number and size of multi-word clusters. All words, including repetitions, were included in semantic analyses.

#### ESA analysis

We computationally analyzed the semantic associations between words using ESA [57]. Pairwise ESA cosines were calculated automatically from a precomputed 155 MB database of word pair associations derived from Wikipedia entries from 2005 (github.com/ticcky/esalib.git). ESA analysis of the “animals” condition showed that the semantic relatedness between successive pairs of words produced by subjects, the pairwise (PW) ESA, ranged from 0.000 (“cockatiel” to “zebra”) to 0.893 (“red-fox” to “gray-fox”). Insofar as word retrieval reflected semantic priming between successive words, we expected higher PW-ESA cosines in comparison to the average ESA cosine (A-ESA) between all of the words retrieved by a subject. In addition, because words belonging to a semantic category (e.g., “animals”) share considerable conceptual similarity, we anticipated higher PW- and A-ESA cosines in semantic conditions than in phonemic conditions.

We also developed a semantic organization index (SOI): the PW-ESA/A-ESA ratio. In semantic testing, this ratio ranged from below 1.0 (for subjects who retrieved animal names in a sequential order that lacked any obvious conceptual basis) to more than 4.0 (for subjects who retrieved animal names in multiple distinct, but tightly related, clusters). We anticipated that SOIs would be higher in semantic than phonemic conditions. However, because of the fundamental semantic organization of verbal memory [88], we hypothesized that some semantic influences (i.e., SOIs above 1.0) would also be evident during phonemic testing [26].

#### ESA analysis of switches and clusters

We categorized ESA switches as PW-ESA values that fell below a fixed percentage of the A-ESA in each subject. The number of ESA switches varied predictably from a mean of 11.45 switches at a threshold of 100% of the A-ESA to a mean of 6.40 switches at a threshold of 50% of the A-ESA. The threshold of 75% of the A-ESA was used for further analysis since it yielded a number of switches (mean = 9.20) that was similar to the number of switches identified with the Troyer method. The number and size of ESA-defined multi-word clusters were also quantified for each subject.

#### Statistical analysis

The results were analyzed with Analysis of Variance (ANOVA) using CLEAVE (www.ebire.org/hcnlab). Greenhouse-Geisser corrections of degrees of freedom were uniformly used in computing p values in order to correct for covariation among factors and interactions, with effect sizes reported as partial ω^2^. Pearson correlation analysis was also used with significance levels evaluated with Student’s t-tests. Linear multiple regression was used to evaluate the contribution of multiple demographic factors on performance and to produce correct-word z-scores.

### Experiment 1: Results

**Fig 1:**
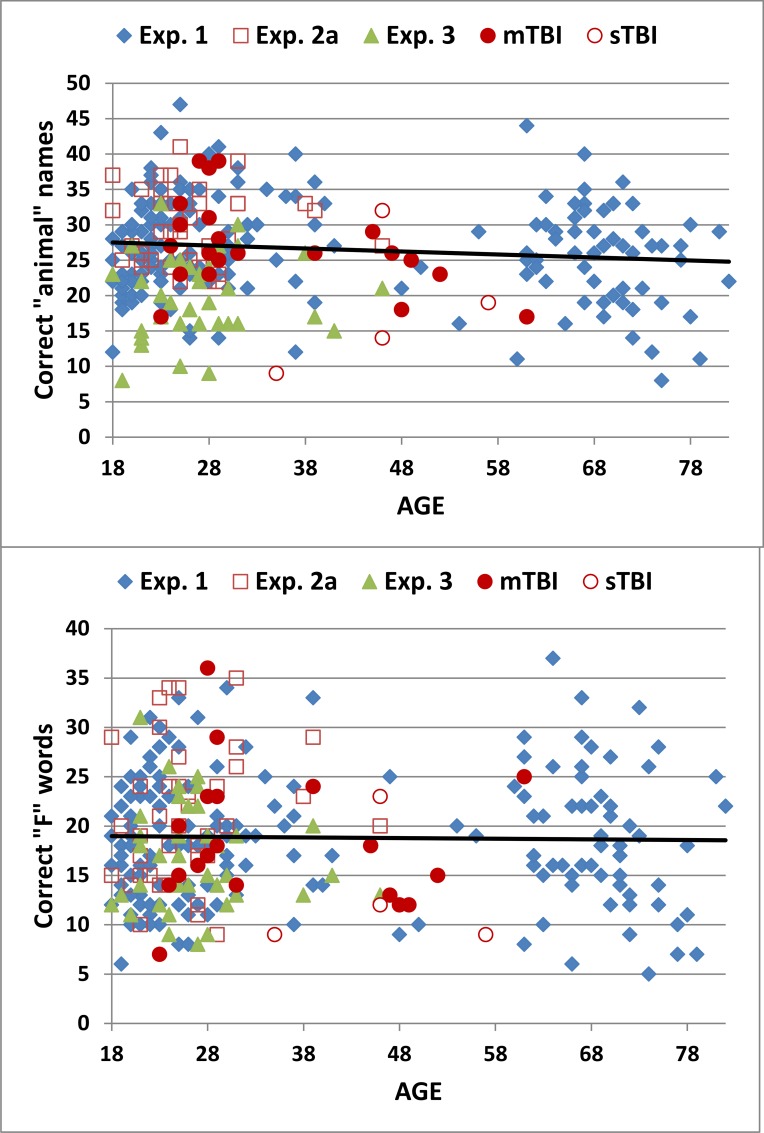
Correct word scores in semantic (“animals”) and phonemic (letter “F”) conditions as a function of age. The data are from Experiment 1, Experiment 2a, Experiment 3 (simulated malingering), and Experiment 4 (mild TBI = mTBI, filled red circles; severe TBI = sTBI, cross-hatched red circles). The age-regression slopes from Experiment 1 are shown.

Fig 1 shows the number of correct words retrieved in semantic (“animal”, top) and phonemic (“F”, bottom) conditions as a function of age for the subjects in Experiment 1 (blue diamonds) and for the subjects in the other experiments discussed below. Figs S1 Fig and S2 Fig show the correct-word scores as a function of education and computer-use.

Subjects retrieved more correct words in semantic than phonemic conditions [26.6 versus 18.8, F(1,179) = 1194.05, p < 0.0001, ω^2^ = 0.52]. Subjects retrieved 20.1 animal names and 14.6 “F” words over the first 60 s of the test; i.e., correct-word scores were similar to the average scores in previous studies using 60 s testing periods (see Table 1). Table 2 provides mean scores of correct words (CW) in semantic and phonemic conditions as well as scores for the other metrics discussed below.

Table 3 and Table 4 show the respective correlation matrices for the semantic and phonemic conditions of Experiment 1. Age had a borderline influence on correct-word scores in semantic conditions [r = -0.13, t(178) = 1.75, p < 0.05, one-tailed], but did not influence correct-word scores in phonemic conditions [r = -0.02, NS]. Sex failed to significantly influence scores in either condition [r = -0.01 and r = -0.12, respectively]. In contrast, Education increased correct-word scores in both semantic [r = 0.31, t(178) = 4.35, p < 0.0001] and phonemic [r = 0.19, t(178) = 2.58, p < 0.02] conditions. We also found significant correlations between computer-use and correct-word scores on both semantic [r = 0.33, t(178) = 4.66, p < 0.0001] and phonemic [r = 0.27, t(178) = 3.74, p < 0.0007] tests.

**Table 3:** Correlation matrix for the semantic (“animals”) condition in experiment 1.

	Edu	C-use	CW	%RW	Syll	LWF	Typ	PW-ESA	A-ESA	SOI	TDP	ESA-Switch	ESA-MWC	Clustersize	Troyer switch
**Age**	0.16	-0.27	-0.13	0.29	-0.08	0.05	-0.05	0.07	0.05	0.06	0.00	-0.09	-0.07	-0.03	-0.13
Edu		0.32	0.31	-0.06	0.03	-0.11	-0.18	-0.01	-0.28	0.25	-0.09	0.27	0.29	-0.09	0.31
**C-use**			0.33	-0.11	0.07	-0.13	-0.11	-0.02	-0.19	0.19	-0.11	0.22	0.30	-0.03	0.30
CW				-0.13	0.09	-0.40	-0.58	0.17	-0.50	0.61	-0.49	0.59	0.73	0.18	0.59
%RW					-0.17	0.14	0.08	-0.05	0.08	-0.10	0.13	0.02	-0.01	-0.04	0.02
Syll						-0.70	-0.27	0.08	0.03	0.08	-0.06	0.28	0.09	-0.14	0.11
LWF							0.69	-0.27	0.14	-0.39	0.37	-0.52	-0.29	0.11	-0.25
Typ								-0.22	0.38	-0.52	0.51	-0.51	-0.44	0.02	-0.36
PW-ESA									0.50	0.64	-0.13	0.02	0.10	0.13	-0.22
A-ESA										-0.26	0.33	-0.30	-0.39	-0.07	-0.49
SOI											-0.40	0.23	0.42	0.23	0.13
TDP												-0.39	-0.26	-0.12	-0.29
ESW													0.61	-0.45	0.67
EMW														-0.42	0.41
ECS															0.05

Abbreviations: ESA = Explicit Semantic Analysis association strength of words in the order produced; A-ESA = ESA association strength for words produced in random order. See Table 2 for additional abbreviations. Given the sample size (N = 180), correlations > |0.15| are significant at p < 0.05, correlations > |0.20| are significant at p < 0.01, correlations > |0.25| are significant at p < 0.001, and correlations > |0.29| are significant at p < 0.0001.

**Table 4:** Correlation matrix for the phonemic (letter “f”) condition in experiment 1.

	Edu	C-use	CW	%RW	Syll	LWF	PW-ESA	A-ESA	SOI	TDP
**Age**	0.16	-0.27	-0.02	0.42	0.19	-0.07	0.02	-0.05	0.04	0.03
Edu		0.32	0.19	0.05	0.00	-0.02	-0.01	-0.15	0.08	-0.14
**C-use**			0.27	-0.19	-0.09	0.04	-0.04	-0.20	0.07	0.03
CWs				-0.14	0.09	-0.04	-0.01	-0.31	0.13	-0.26
%RWs					0.11	-0.10	0.01	0.08	-0.04	0.01
Syll						-0.07	0.02	-0.17	0.10	-0.01
LWF							0.15	0.19	0.09	0.04
PW-ESA								0.56	0.84	0.11
A-ESA									0.10	0.13
SOI										0.05

See Tables 2 and 3 for abbreviations.

Multiple regression with Age, Education, and Computer-use as factors accounted for 17.0% of the variance in semantic conditions and 8.5% of the variance in phonemic conditions. The contribution of Age to the multiple regression was not significant in either condition. However, Education and Computer-use made significant, independent contributions in semantic conditions [respectively, t(176) = 3.51, p < 0.0006 and t(176) = 2.81, p < 0.006]. In the phonemic condition, the independent contribution of Education only approached significance, while the influence of Computer-use persisted [t(176) = 3.04, p < 0.003]. Correct-word z-scores were derived after regressing out the influence of Education and Computer-use using the equation CW = 10.61 + 0.781*Education + 0.912*Computer-use for “animal” fluency, and the equation CW = 9.65 + 0.356*Education + 0.789*Computer-use for letter “F” fluency.

The percentage of repeated words was small in the semantic condition (1.28%), with only 20% of subjects producing repetitions. Repetitions were more frequent (2.61%) in the phonemic condition [F(1,179) = 11.11, p < 0.001, ω^2^ = 0.05], with 31% of subjects producing repetitions. Older subjects produced more repetitions than younger subjects, resulting in significant correlations between age and the percentage of repetitions in semantic [r = 0.29, t(178) = 4.04, p < 0.0001] and phonemic [r = 0.42, t(178) = 6.17, p < 0.0001] conditions.

**Fig 2:**
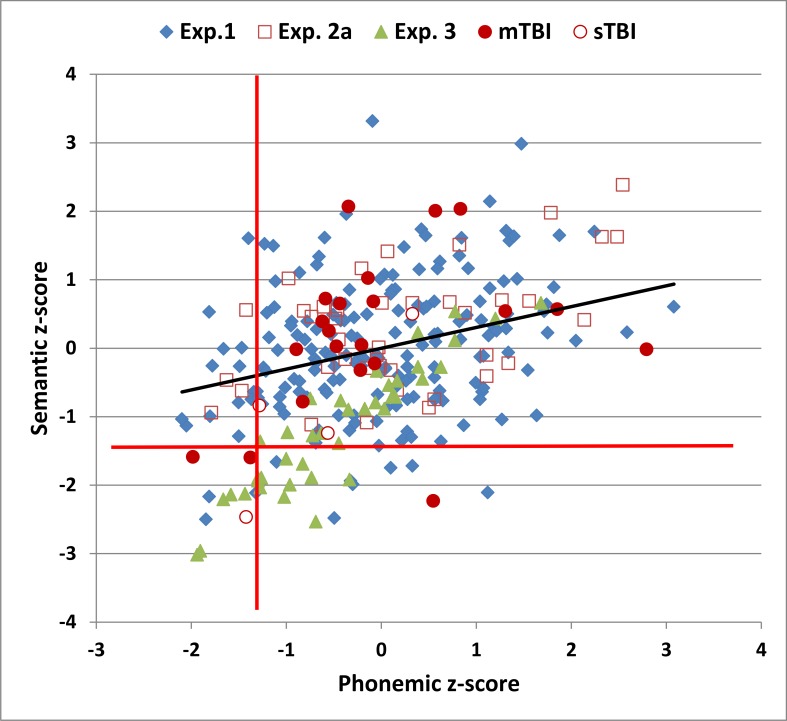
Correct-word z-scores in semantic and phonemic conditions. Z-scores were derived after adjusting for education and computer-use using the regression equation from Experiment 1. The horizontal and vertical red lines show abnormality thresholds (p < 0.05) in semantic and phonemic conditions, respectively. The regression line is from the data in Experiment 1. See Fig 1 for further details.

Fig 2 plots the correct-word z-scores of individual subjects (blue diamonds) in semantic versus phonemic conditions. Significant correlations were seen between semantic and phonemic z-scores [r = 0.30, t(178) = 4.20, p < 0.0001], correct-word scores [r = 0.38, t(178) = 5.48, p < 0.0001], and the percentage of repeated words [r = 0.42, t(178) = 6.17, p < 0.0001].

#### Temporal analysis

**Fig 3:**
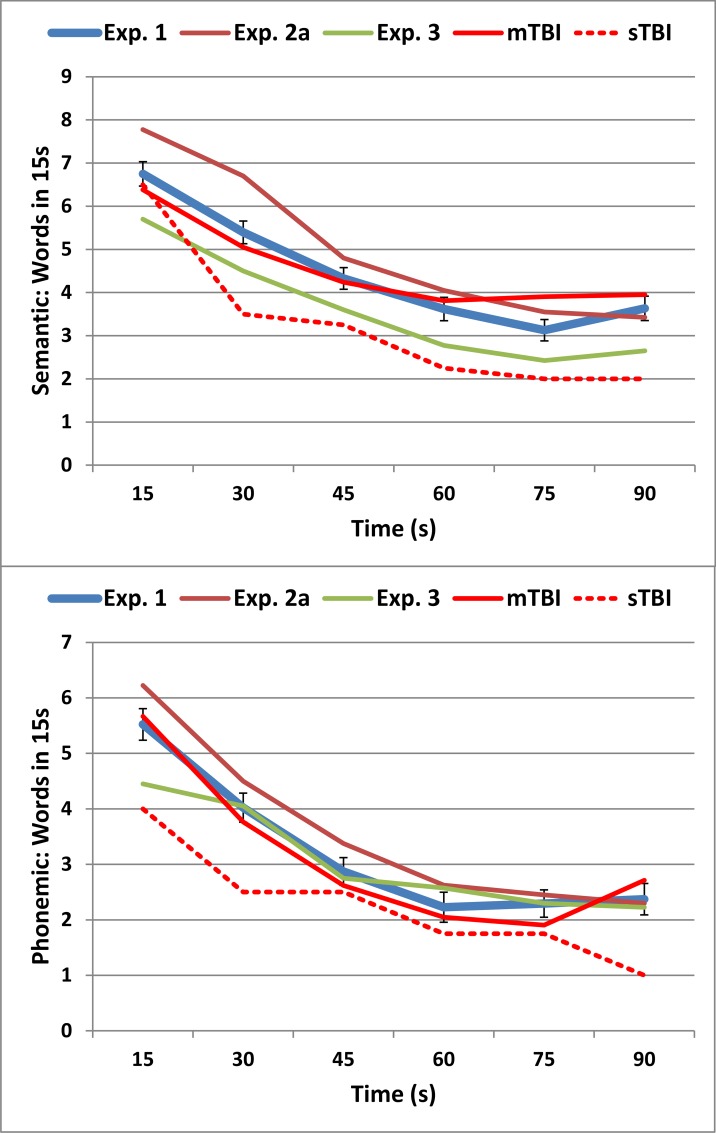
Rate of word production over 15 s intervals in semantic (top) and phonemic (bottom) conditions. Error bars show standard errors for Experiment 1 data. See Fig 1 for further details.

Fig 3 shows the rate of word production for each 15 s interval in the semantic (top) and phonemic (bottom) conditions of Experiment 1 (thick blue line). The word production rate declined sharply, with the temporal decline percentage (TDP) averaging 62.6% in semantic conditions and 65.3% in phonemic conditions. The TDP was slightly greater in phonemic than semantic tests [F(1,179) = 5.79, p < 0.02, ω^2^ = 0.03]. Subjects with increased TDPs showed reduced correct-word scores in both semantic [r = -0.49, t(178) = -7.50, p < 0.0001] and phonemic [r = -0.26, t(178) = -3.59, p < 0.0005] conditions.

#### Lexical analysis

Subjects used more frequent words in phonemic than semantic tests [F(1,179) = 660.03, p < 0.0001, ω^2^ = 0.79]. As expected, subjects with lower correct-word scores used more frequent words in both conditions [semantic: r = -0.40, t(178) = -5.82, p < 0.0001; phonemic: r = -0.34, t(178) = -4.82, p < 0.0001]. Word syllable counts were also greater in “animal” than “F” conditions [F(1,179) = 180.43, p < 0.0001, ω^2^ = 0.50], and were negatively correlated with word frequency in both conditions [semantic, r = -0.71, t(178) = -13.45, p < 0.0001; phonemic, r = -0.43, t(178) = -6.35, p < 0.0001]. However, syllable counts did not significantly correlate with correct-word scores in either test.

Word typicality was only analyzed in semantic conditions. Typicality scores showed strong correlations with correct-word scores [r = -0.58, t(178) = -9.45, p < 0.0001] and word frequency [r = 0.69, t(178) = 12.72, p < 0.0001]. Post-hoc analysis showed that the correct-word scores had a stronger correlation with word typicality than word frequency [z = 2.25, p < 0.03]. In addition, typicality scores correlated strongly with the TDP [r = 0.51, t(178) = 7.91, p < 0.0001]; i.e., subjects who produced more typical words showed a more rapid temporal decline in retrieval.

#### Semantic analysis

In the semantic condition, PW-ESA cosines were more than twice as large as A-ESA values, producing a mean SOI of 2.07. As shown in Table 3, PW-ESA and A-ESA measures were positively correlated with each other [r = 0.50, t(178) = 7.70, p < 0.0001]. PW-ESA measures showed a positive correlation with correct-word scores [r = 0.17, t(178) = 2.30, p < 0.03] while A-ESA measures showed a more substantial negative correlation [r = -0.50, t(178) = -7.70, p < 0.0001].

**Fig 4:**
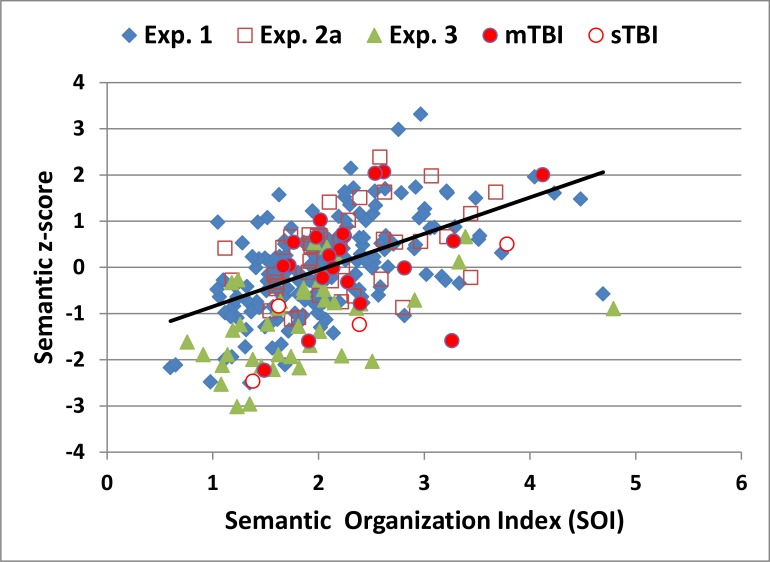
Correct word z-scores and semantic organization indices (SOIs) in the semantic (“animals”) conditions. The regression line is from Experiment 1. See Fig 1 for further details.

Fig 4 shows the relationship between correct-word z-scores and SOIs for the subjects in Experiment 1 (blue diamonds) and subsequent experiments. The correlation of SOIs with correct-word scores [r = 0.61, t(178) = 10.27, p < 0.0001] indicates that subjects who retrieved successive words that were closely related (i.e., a high PW-ESA) in distinct semantic categories (i.e., a low A-ESA) retrieved more correct words. The SOI also showed significant negative correlations with typicality [r = -0.52, t(178) = -8.12, p < 0.0001], indicating that subjects who used more typical words had poorer semantic organization, and with the TDP [r = -0.40, t(178) = -5.82, p < 0.0001], indicating that subjects with better semantic organization retrieved more words later in the test.

All measures of semantic relatedness were predictably reduced in phonemic conditions compared to semantic conditions, including the PW-ESA [F(1,179) = 346.71, p < 0.0001, ω^2^ = 0.66], the A-ESA [F(1,179) = 820.52, p < 0.0001, ω^2^ = 0.82], and the SOI [F(1,179) = 82.41, p < 0.0001, ω^2^ = 0.31]. Comparisons between semantic and phonemic tests showed no significant across-condition correlations for either the PW-ESA or SOI, and only a minimal correlation for the A-ESA [r = 0.15, t(178) = 2.02, p < 0.05].

Nevertheless, the SOI significantly exceeded 1.0 [mean = 1.39, F(1,179) = 40.86, p< 0.0001, ω^2^ = 0.18] in phonemic tests, indicating a significant semantic influence on the order of words retrieved despite the explicitly phonemic nature of the task. The PW-ESA was not significantly correlated with correct-word scores in phonemic conditions. In contrast, there was a significant negative correlation between correct-word scores and the A-ESA [r = -0.31, t(178) = -4.35, p < 0.0001]; i.e., subjects who retrieved words in more distinct semantic categories during phonemic testing produced more correct words.

#### Semantic analysis: switches and clusters

Switches and clusters were only examined in semantic conditions. Subjects produced an average of 9.18 ESA-defined switches and 12.10 Troyer-defined switches. The number of ESA- and Troyer-defined switches correlated strongly in individual subjects [r = 0.67, t(178) = 12.04, p < 0.0001]. Moreover, individual words classified as Troyer switches were often classified as ESA switches [r = 0.41, Χ^2^(1) = 813.6, p < 0.0001].

As shown in Table 3, both the number of ESA- and Troyer-defined switches correlated strongly with correct-word scores [r = 0.59, t(178) = 9.75, p < 0.0001]. The number of switches was not significantly correlated with age for either measure, but the number of switches increased in subjects with greater education [ESA: r = 0.27, t(178) = 3.74, p < 0.0003; Troyer: r = 0.31, t(178) = 4.35, p < 0.0003] and computer-use [ESA: r = 0.22, t(178) = 3.00, p < 0.004; Troyer: r = 0.30, t(178) = 4.20, p < 0.0001].

The number of ESA- and Troyer-defined switches correlated strongly with lexical measures, including log word frequency and typicality [p<0.0001 for all comparisons]. ESA, but not Troyer switches, also correlated significantly with syllable count [p< 0.0002]. Subjects who produced more switches showed reduced TDPs [p<0.0001 for both comparisons]. In general, statistical comparisons of correlation coefficients showed that ESA switches correlated more strongly with lexical and temporal measures than Troyer switches [p< 0.003 to p< 0.09 for the different comparisons].

The number of multi-word clusters (mean ESA = 5.72, Troyer = 6.31) showed the strongest correlation of any metric with correct-word scores [r = 0.73, t(178) = 14.25, p < 0.0001 for both ESA and Troyer methods]. The number of multi-word clusters also correlated significantly with education [ESA, p < 0.0001; Troyer, p < 0.002], computer-use [ESA: p < 0.0001; Troyer: p < 0.004], and lexical factors including log word frequency [ESA: p < 0.0003; Troyer: p < 0.005] and typicality [p < 0.0001 for both comparisons].

Cluster size (mean ESA = 4.06 words, Troyer = 3.21 words) also correlated significantly with correct-word scores [ESA: p < 0.02; Troyer: p < 0.0001] and the SOI [ESA: p < 0.002; Troyer: p < 0.0001]. Cluster size was not significantly correlated with age, education, or computer-use for either method.

### Experiment 1: Discussion

The subjects in Experiment 1 produced correct-word scores in semantic and phonemic conditions that were in the mid-range of scores reported in previous large-scale studies (see Table 1). In semantic conditions, correct-word scores correlated more weakly with age than in many previous studies [18, 59, 71], presumably in part because of the high mean education level of our older subject population. Consistent with previous studies, age correlations were further reduced in phonemic conditions [64]. However, we found a moderately strong age-related increase in the percentage of repeated words in both conditions [66].

As in previous studies, we found significant effects of education on correct-word scores [18, 59, 62, 71]. In addition, we found a significant relationship between computer-use and correct-word scores that persisted after the effects of education had been factored out. These results suggest that computer-use, like education, is a useful supplementary demographic correlate of VF performance. There are two possible explanations for this correlation. First, IQ may correlate with computer use. To evaluate this hypothesis, we examined the correlation between computer-use and scores on the Wechsler Test of Adult Reading (WTAR), which correlates strongly with measures of IQ [89, 90]. We found that computer-use was significantly correlated with WTAR scores [r = 0.25 t(175) = 4.01, p <0.0001], and this correlation remained significant after the effect of education had been factored out [t(174) = 2.66, p< 0.01]. Second, subjects who read with computers may benefit from the embedded links in computer text that connect related topics. For example, the Wikipedia entry for “dog” provides links to related species (e.g., wolves, jackals, coyotes, etc.) and different dog breeds. As a result, computer links may strengthen semantic associations.

#### Temporal and lexical analysis

Word production rates declined throughout the test [25], and subjects with increased TDPs showed reduced correct-word scores [91]. Word frequencies were greater and syllable counts were reduced in phonemic conditions compared to semantic conditions [25]. Word frequencies and word typicality showed predictably negative correlations with correct-word scores in semantic conditions [29], with post-hoc analysis showing that correct-word scores were more strongly correlated with word typicality than word frequency. Subjects who used more frequent and typical words also showed a greater temporal decline in word production.

#### Semantic analysis

In semantic conditions, the association between successively retrieved words (PW-ESA) was more than twice as strong as the average associations among all words retrieved (A-ESA). The PW-ESA/A-ESA ratio was used to create a semantic organization index (SOI), which summarized the degree of semantic ordering of retrieval for each subject. Subjects with greater correct-word scores showed higher SOIs, suggesting that they were able to retrieve related words from more distinct semantic categories.

Explicit Semantic Analysis revealed predictably stronger semantic associations between words in semantic than phonemic conditions. However, in phonemic conditions, the SOI significantly exceeded 1.0, revealing significant semantic influences on the order of words reported despite the explicit phonemic nature of the task [26].

#### Switches and clusters

We quantified switches and clusters in semantic conditions using both Troyer [39] and ESA methods. The subjects in Experiment 1 produced 20.1 words and 9.2 Troyer-defined switches over 60 s, similar to the 19.5 words and 9.8 switches observed in the normative study of Troyer (2000) [43]. Although the number of Troyer-defined switches exceeded the number of ESA-defined switches, words identified as Troyer switches were often identified as ESA switches (r = 0.41).

As in previous studies [39], the number of correct words correlated strongly with the number of semantic switches measured with both methods. This reflects in large part the arithmetic relationship between the number of switches and the number of words retrieved: a subject with N switches would necessarily retrieve at least N+1 words. An even stronger correlation was found between correct words and the number of multi-word clusters, reflecting the fact that a subject who produces retrieves N multi-word clusters would necessarily retrieve at least 2*N words. As in previous studies, the size of multi-word clusters was only weakly correlated with correct-word scores [50, 52].

We found strong correlations between semantic and lexical measures. For example, the SOI, the number of switches, and the number of multi-word clusters all showed negative correlations with word frequency and word typicality, implying that subjects with better semantic organization retrieve less frequent and less typical words. Moreover, the SOI, number of switches, and number of multi-word clusters were all negatively correlated with temporal decline. Thus, subjects with better semantic organization were able to sustain effective word retrieval later in the test.

#### Differences between switch and SOI measures

The SOI reflects the ability of subjects to retrieve semantically related words in sequence (as reflected in a high PW-ESA) from distinct regions of semantic memory (as reflected in a low A-ESA). Unlike the number of switches and multi-word clusters, the SOI is not computationally related to correct-word scores. For example, in a subject who retrieved 16 words in four tightly related, but highly distinct four-word clusters (e.g., “Doberman, German Shepard, Rottweiler, Mastiff; Holstein, Angus, Brahma bull, Charolais; Red tailed hawk, Cooper’s hawk, Bald Eagle, Osprey; Tarantula, Black widow, Jumping spider, Wolf spider”), the PW-ESA would be high and the A-ESA low, resulting in a high SOI despite a low correct-word score, only three switches and three multi-word clusters. However, if the subject retrieved the same 16 words in clusters of two words each, the A-ESA would remain unchanged, but the PW-ESA, and hence the SOI, would be reduced while the number of semantic switches and multi-word clusters would double.

#### Semantic switches identified with ESA and Troyer methods

More words were identified as Troyer switches than ESA switches, in part reflecting the occasional strong semantic associations between words in different Troyer subcategories (e.g., “tiger” and “shark”). The increased number of Troyer switches may also reflect the non-exhaustive nature of Troyer subcategories. For example, there is no Troyer subcategory for Latin American animals. Hence, a subject retrieving South American animals (e.g., “howler monkey, tapir, llama, spider monkey, piranha, ocelot, harpy, etc.”) would produce more Troyer switches than ESA switches. Conversely, ESA would generally identify more switches than the Troyer method when words occurred in multiple Troyer subcategories. For example, in the hypothetical sequence described above with no Troyer switches (“rabbit, cat, tiger, lion, zebra, crocodile, whale”), ESA would typically identify switches between “rabbit” and “cat”, “zebra” and “crocodile”, and “crocodile” and “whale”.

The fact that ESA switches were defined by an arbitrary cutoff (e.g., 75% of the A-ESA) has another important consequence: unlike Troyer methods, ESA will almost always identify semantic switches in the list of words retrieved. For example, in a subject who reports only dog breeds (e.g., “Dachshund, Great Dane, Chihuahua, Pug, Pekingese, Corgi, Basset, Beagle, Weimaraner, German Shepard, Australian Shepard, Border Collie, Rottweiler, Pit Bull, Staffordshire, Wolfhound, Deerhound”), ESA switches would be identified between different breed types (e.g., companion dogs, hunting dogs, etc.) and between dogs of different size. In contrast, no Troyer switches would occur because all animals are both pets and canids there are no Troyer switches.

This example highlights another difference between ESA and Troyer methods: the determination of whether a word pair is an ESA switch depends on the other words retrieved. Thus, a word pair (e.g., “Dachshund” and “Great Dane”) would be an ESA switch in a subject who names only dog breeds, but would be clustered together in another subject who names a many different types of animals. The context sensitivity of ESA makes it possible to apply to categories of different size (e.g., “animals”, “pets”, “breeds of dog”).

#### Limitations

A larger and more demographically varied subject population is needed to ensure that the C-VF norms reported here are suitable for general use. In particular, our older subjects were very well educated, which likely minimized age-related decline in correct-word scores [18, 71, 92].

### Experiment 2: Generalization of Normative Data and Test-Retest Reliability

Experiment 2 analyzed the results of repeated C-VF testing in 40 young and well-educated subjects who were tested three times at weekly intervals. The first test session (Experiment 2a) used the same two categories (“animals” and the letter “F”) as Experiment 1, while Experiments 2b and 2c used different semantic categories and different letters.

We focused on two aspects of the results. First, we evaluated the extent to which the results from Experiment 1 would generalize to a population of younger and somewhat better educated subjects in Experiment 2a. We anticipated that the subjects in Experiment 2a would retrieve more correct words than the subjects in Experiment 1, but would show similar correct-word z-scores after the contributions of education and computer-use had been factored out using the regression functions derived in Experiment 1.

Second, we were interested in the test-retest reliability of C-VF measures. High test-retest reliabilities have been previously reported for correct-word scores in phonemic tests using different letters [18, 93, 94] (i.e., intraclass correlation coefficients, ICCs, above 0.75), along with significant differences in the number of correct words retrieved (S>F>A) [18]. In contrast, the percentage of repeated words has shown relatively low test-retest correlations (r < 0.25) [53, 95].

Although significant differences have also been noted in correct-word scores for different semantic categories (e.g., “animals” > “professions”) [59], the test-retest reliability of correct-word scores when tests use different semantic categories has not previously been investigated. We anticipated lower correlations between correct-words scores in semantic than phonemic conditions since semantic fluency would likely be influenced to a greater degree by the different interests and hobbies of subjects. For example, some subjects may have been members of local zoological societies (proficient in the “animals” category), whereas others may have been amateur chefs (proficient in the “foods” category).

The test-retest reliability of lexical, temporal, and semantic measures of VF performance have not been studied in detail. Indeed, to our knowledge, no previous studies have examined the test-retest reliability of word frequency, word length, or temporal changes in response rate over time. Furthermore, the test-retest reliability of Troyer-defined switches and clusters in semantic fluency tests has not been established, either with repeated tests in the “animals” category or when different semantic categories are used.

### Experiment 2: Methods

#### Subjects

Forty young volunteers (mean 25.8 years, range 18–46 years, 53% male) were recruited primarily from online advertisements on Craigslist. Subjects who met the same inclusion criteria listed in Experiment 1 volunteered to participate in three weekly test sessions. As seen in Table 2, subjects were primarily college students who were significantly younger [p < 0.01] and reported higher levels of computer-use [p< 0.03] than the subjects in Experiment 1. Ethnically, 68% of the subjects were Caucasian, 11% Latino, 9% African American, 10% Asian, and 2% “other”.

#### Procedures

The test administration methods were identical to those described in Experiment 1. In Experiments 2a, 2b, and 2c, the semantic categories were respectively “animals”, “parts of the body”, and “foods”, and the phonemic categories were “F”, “A”, and “S”. The order of the categories was identical for every subject. Because Troyer subcategories have not yet been defined for “parts of the body” and “foods”, we did not perform Troyer analyses.

#### Statistical analysis

The results were analyzed with the methods used in Experiment 1, while intraclass correlation coefficients (ICCs) were analyzed with SPSS (version 25).

### Experiment 2: Results

Table 2 includes summary performance means and standard deviations from the three test sessions in Experiment 2 (2a, 2b, and 2c). Fig 1 includes the correct-word scores from subjects in Experiment 2a (open red squares), Fig 2 shows the correct-word z-scores of individual subjects in Experiment 2a, and Fig 3 shows the rate of word production in Experiment 2a (dark red lines).

An ANOVA analysis of scores with Group (Experiment 1 vs Experiment 2a) and Test-type (semantic and phonemic) as factors showed a Group effect [F(1,218) = 6.71, p < 0.02, ω^2^ = 0.03] due to greater correct-word scores in Experiment 2a. There was also a large effect of Test-type [F(1,218) = 259.66, p < 0.0001, ω^2^ = 0.54] due to more correct words in semantic than phonemic conditions, but no significant Group x Test-type interaction [F(1,218) = 0.35, NS]. After correcting scores for education and computer-use using the regression equations from Experiment 1, the effects of Group [F(1,218) = 3.23, p < 0.10] and Test-type [F(1,218) = 0.03, NS] were no longer significant.

Comparisons between Experiment 1 and Experiment 2a showed no significant differences in the SOI [F(1,218) = 2.09, NS], the TDP [F(1,218) = 1.39, NS], the number of syllables [F(1,218) = 0.53, NS], or word frequency [F(1,218) = 0.15, NS]. However, the subjects in Experiment 2a had lower typicality scores than those in Experiment 1 [F(1,218 = 34.48, p< 0.001, ω^2^ = 0.13]. In addition, the number of ESA switches in the semantic condition was increased in Experiment 2a compared to Experiment 1 [F(1,218) = 5.07, p < 0.03, ω^2^ = 0.02], as was the number of multi-word clusters [F(1,218) = 4.86, p < 0.03, ω^2^ = 0.02], without significant differences in cluster size [F(1,218) = 0.24, NS].

A comparison of correct-word scores across the three test sessions of Experiment 2 (Table 2) revealed significant differences as a function of semantic [F(2,78) = 10.31, p < 0.0001, ω^2^ = 0.19] and phonemic [F(2,78) = 40.98, p < 0.0001, ω^2^ = 0.51] categories. In semantic conditions, subjects retrieved more words in “body parts” (Experiment 2b) and “foods” (Experiment 2c) than in “animals” (Experiment 2a). In phonemic conditions, subjects retrieved more words beginning with the letter “S” (Experiment 2c) than the letter “F” (Experiment 2a), and more words beginning with the letter “F” (Experiment 2a) than the letter “A” (Experiment 2b).

As in Experiment 1, SOIs were universally higher in semantic than phonemic tests, and more frequent words were used in phonemic than semantic conditions. The mean number of syllables was also greater in semantic than phonemic tests with one exception: syllable counts were increased in letter “A” testing, presumably because few single-syllable words begin with vowels.

Further analysis of the three semantic conditions showed significant differences in the SOI [F(2,78) = 5.03, p < 0.01, ω^2^ = 0.09] (increased in foods), the number of switches [F(2,78) = 6.93, p < 0.003, ω^2^ = 0.13] (increased in foods), and cluster size [F(2,78) = 4.03, p < 0.03, ω^2^ = 0.07] (increased in body parts). No significant differences were seen in the number of multi-word clusters [F(2,78) = 0.19, NS]. There were also significant differences across categories in the TDP [F(2,78) = 11.93, p < 0.0001, ω^2^ = 0.20] (reduced in foods).

**Fig 5:**
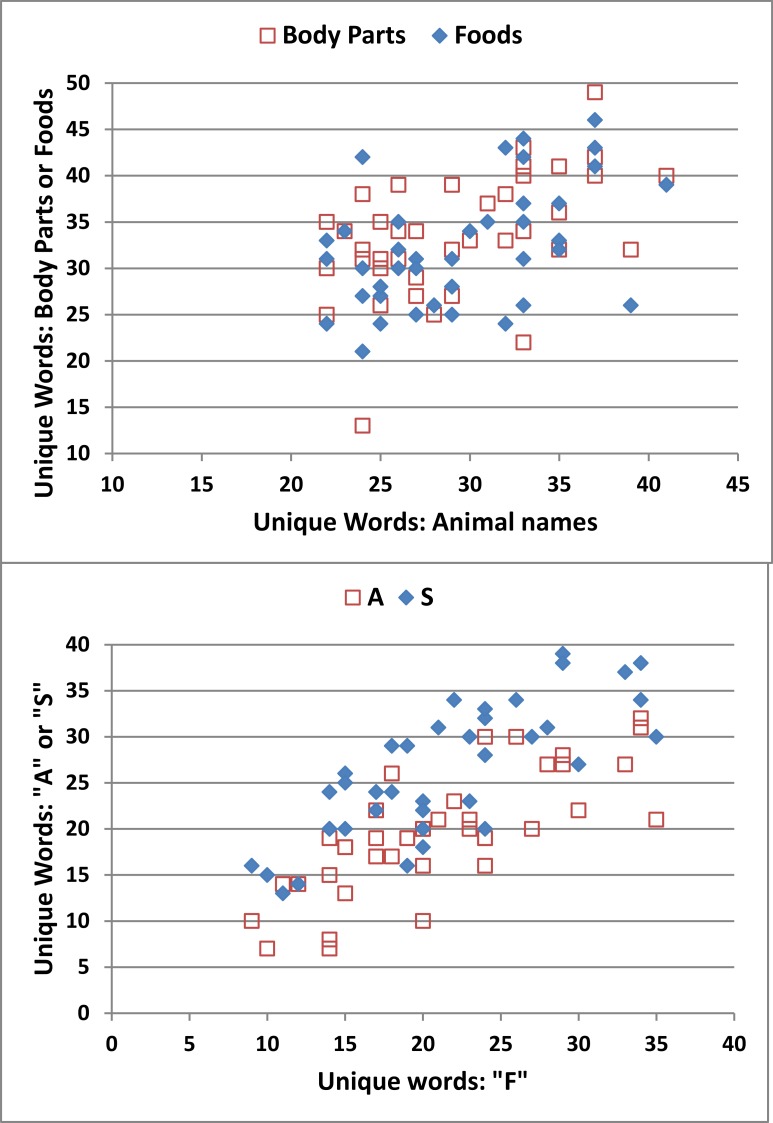
Correct-word scores in different semantic (top) and phonemic (bottom) tests in Experiment 2. Semantic conditions were “animals”, “parts of the body”, and “foods”. Phonemic conditions were the letters “F”, “A”, and “S”.

As shown in Fig 5, strong correlations were evident in correct-word scores across semantic (top) and phonemic conditions (bottom). Table 5 shows the ICCs for the different metrics. The highest ICCs were seen for correct-word scores: 0.77 in semantic conditions and 0.91 in phonemic conditions. A statistical comparison of the two ICCs showed that the correlations were significantly stronger in phonemic than semantic conditions [z = -4.77, p < 0.0001].

**Table 5:** Test-retest reliability of different measures in semantic and phonemic conditions of experiment 2.

	CW	%RW	LWF	Syll	PW-ESA	A-ESA	SOI	ESW	EMW	ECS	TDP
Semantic	0.77	-0.02	0.48	0.13	0.56	0.43	0.68	0.62	0.47	-0.27	0.31
Phonemic	0.91	0.50	0.05	0.69	0.31	0.25	0.44				0.54
Mean: S vs P	0.70	0.18	0.08	0.41	-0.02	0.28	0.27				0.05

The top two lines show the intraclass correlation coefficients (ICCs) across the three semantic and three phonemic tests. Mean S vs. P: the Pearson correlations between average scores in semantic and phonemic conditions. Different categories and different letters were used in each test. Given the sample size (40), correlations exceeding 0.32 were significant at the p < 0.05 level, one-tailed. See Tables 2 and 3 for abbreviations.

**Fig 6:**
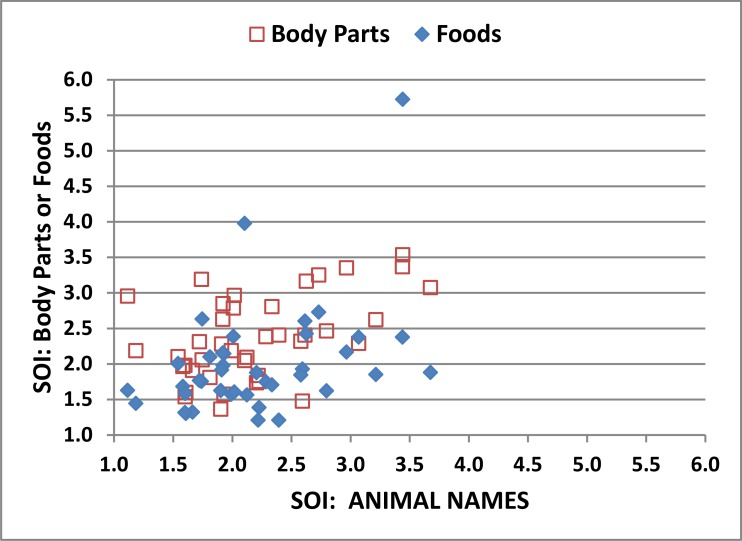
Semantic organization index (SOI) for different semantic categories. Data from Experiment 2 showing the relationship between SOIs produced in different semantic categories, “animals” (ordinate) and “foods” and “body parts” (abscissa).

As shown in Fig 6, the SOI also showed good reliability in semantic conditions (ICC = 0.68), with significant reliability seen for both PW-ESA and A-ESA measures (see Table 5). This suggests that both the strength of semantic priming and the extent of semantic space explored were characteristic of individual subjects. In addition, ESA-defined semantic switches showed an ICC of 0.62 over different categories, while the number of multi-word clusters (ICC = 0.47) showed lower, but still highly significant, reliability. Finally, syllable counts in semantic conditions, the number of words in multi-word clusters, and the percentage of repeated words showed insignificant ICCs.

In contrast, in phonemic conditions both the percentage of repeated words and word syllable counts showed significant correlations across different letters. The SOI also showed significant correlations, indicating that semantic influences were consistent in different phonemic tasks. Finally, the TDP showed a significant correlation, indicating a consistent pattern of temporal decline in phonemic word retrieval.

Further comparisons between semantic and phonemic conditions showed strong correlations in overall correct-word scores [r = 0.70, t(38) = 6.04, p < 0.0001] and a significant correlation in word syllable counts [r = 0.41, t(38) = 2.77, p < 0.01], but non-significant correlations for the other metrics.

### Experiment 2: Discussion

The slightly better educated and more computer literate subjects in Experiment 2 retrieved more correct words in the “animals” and “F” conditions than the subjects in Experiment 1, as well as more switches and multi-word clusters. However, when correct-word scores were transformed into z-scores by factoring out the influences of education and computer-use, inter-group differences lost statistical significance. This indicates that the regression functions developed in Experiment 1 generalized to the younger and somewhat better educated population in Experiment 2. Other differences in performance scores with Group 1 subjects were not significant, with the exception that less typical words were used by the subjects in Experiment 2a.

We found differences in the average number of words retrieved in different semantic and phonemic categories. Similar differences have been found between semantic categories in previous studies [59]. In phonemic conditions, the relative ranking of correct-word scores in “FAS” testing was similar to that reported by Tombaugh et al. [18]. Performance in phonemic testing may have also been influenced by learning effects, since test order was fixed [96]. However, only minimal learning effects have been found in semantic fluency tests, even when identical categories are used for repeated testing [95].

High test-retest reliability of correct-word scores was seen across phonemic tests with different letters, with ICCs (0.91) that significantly exceeded those (0.77) for different semantic categories. This result is unsurprising, since a subject’s experience with different semantic categories is likely to vary more substantially than their exposure to different letters. For example, gender differences in semantic experience likely contribute to male vs. female differences in correct-word scores for different categories [23, 59, 72]. Test-retest reliability of correct-word scores in phonemic testing was somewhat higher than that reported in previous studies [53], perhaps due to the longer duration of the C-VF test (90 s vs. the typical 60 s test) and the relatively short test-retest intervals (one week).

#### The reliability of ESA measures of semantic organization

In semantic conditions, the SOI showed high test-retest reliability (ICC = 0.68), indicating that it is a reliable characteristic of individual subjects when tested with different semantic categories. Highly significant correlations were also seen for the number of semantic switches and the size of ESA-defined multiword clusters. The strong correlation between ESA measures in semantic conditions and correct-word scores indicates that semantic organization is an important determinant of word retrieval, regardless of category.

### Experiment 3: The Effects of Simulated Malingering

When a patient’s neuropsychological test results fall into the abnormal range, the examiner is faced with the challenge of determining whether impaired performance is due to organic causes or suboptimal effort. Previous studies have shown that subjects instructed to malinger retrieve fewer correct words on VF tests than subjects performing with full effort, with word scores in simulated malingering conditions falling roughly one standard deviation below full-effort scores [96]. Other studies have found reductions in correct-word scores of similar magnitude in clinical samples thought to be malingering [97, 98], and noted increases in the incidence of repeated words [97].

### Experiment 3: Methods

#### Subjects and Procedures

The methods were identical to those used in Experiment 1, except for the instructions given prior to testing. All of the 40 subjects had previously completed Experiment 2. As in Experiments 1 and 2a, the subjects were tested with the semantic category “animals” and the phonemic category “F”. They had been given written instructions to perform like a patient with a minor head injury one week prior to Experiment 3. The additional instructions were as follows: “Listed below you’ll find some of the symptoms common after minor head injuries. Please study the list below and develop a plan to fake some of the impairments typical of head injury when you take the next test. Do your best to make your deficit look realistic. If you make too many obvious mistakes, we’ll know you’re faking! Symptom list: Difficulty concentrating for long periods of time, easily distracted by unimportant things, headaches and fatigue (feeling “mentally exhausted”), trouble coming up with the right word, poor memory, difficulty performing complicated tasks, easily tired, repeating things several times without realizing it, slow reaction times, trouble focusing on two things at once.”

#### Statistical analysis

The results were analyzed with Analysis of Variance (ANOVA) using CLEAVE (www.ebire.org/hcnlab) and Greenhouse-Geisser corrections of degrees of freedom. Of primary interest were comparisons with Experiment 1 and Experiment 2a results.

### Experiment 3: Results

Table 2 includes summary performance statistics from Experiment 3. Fig 1 shows the correct-word scores from individual simulated malingering subjects (green triangles), and Fig 2 shows their correct-word z-scores in semantic and phonemic conditions. In comparison with the subjects in Experiment 1, simulated malingerers showed reduced correct-word z-scores in semantic [-1.18, F(1,218) = 45.47, p < 0.0001, ω^2^ = 0.17] and phonemic [-0.46, F(1,218) = 7.00, p < 0.01, ω^2^ = 0.03] conditions. There was also an increase in repeated words in both conditions [F(1,218) = 67.32, p < 0.0001, ω^2^ = 0.24; and F(1,218) = 37.70, p < 0.0001, ω^2^ = 0.15, respectively]. Correct-word scores in simulated malingerers were also reduced relative to their performance in Experiment 2a in both semantic [F(1,39) = 61.35, p < 0.0001, partial ω^2^ = 0.61] and phonemic [F(1,39) = 13.19, p < 0.001, partial ω^2^ = 0.24] conditions. In semantic conditions, 38% of simulated malingerers showed abnormal (p < 0.05) correct-word z-scores, while 15% had abnormal results in phonemic conditions.

Fig 3 shows the rate of word production in both conditions for the subjects in Experiment 3 (green lines). In semantic conditions, the word retrieval rate declined in parallel with that of the subjects in Experiment 1, but with reduced retrieval during each 15 s interval. In contrast, retrieval rates in phonemic conditions were similar in full-effort and malingering conditions, except for the initial 15 s period.

In the semantic condition the number of ESA-defined switches was significantly reduced in comparison with Experiment 1 [F(1,218) = 9.50, p < 0.005, ω^2^ = 0.04] and Experiment 2a [F(1,39) = 19.47, p < 0.0001, partial ω^2^ = 0.32], as was the number of multi-word clusters [F(1,218) = 18.48, p < 0.0001, ω^2^ = 0.07; F(1,39) = 34.48, p < 0.0001, partial ω^2^ = 0.46], without a significant alteration in multi-word cluster size.

Fig 4 shows the relationship between correct-word z-scores and the SOI in simulated malingerers (green triangles) in semantic conditions. Surprisingly, there was no significant reduction in the SOI in simulated malingerers compared to Experiment 1 subjects [F(1,218) = 0.13, NS], although there was a small reduction in comparison to their performance in Experiment 2a [F(1,39) = 5.55, p < 0.03, partial ω^2^ = 0.10]. Typicality showed a trend towards an increase versus Experiment 1 [F(1,218) = 3.48, p < 0.07], and was significantly increased relative to Experiment 2a [F(1,39) = 17.22, p < 0.0002, partial ω^2^ = 0.29]. Word frequencies did not differ from Experiment 1 [F(1,218) = 1.08, NS], but were reduced with respect to Experiment 2a [F(1,39) = 14.22, p < 0.0005, partial ω^2^ = 0.25], while syllable counts showed no significant overall change with respect to either control condition.

Table 6 shows the results from the nine control subjects (top) and 15 malingering subjects (middle) with abnormal correct-word z-scores in semantic conditions. Z-score cutoffs were relatively ineffective in distinguishing abnormal malingerers from control subjects with abnormal scores. For example, a z-score cutoff of -2.0 provided 66% sensitivity and 40% specificity, a cutoff of -2.5 provided 20% sensitivity and 88% specificity, and a cutoff of -3.0 provided 7% sensitivity and 100% specificity.

**Table 6:** Malingering indices in subjects with abnormal semantic fluency z- scores.

Subject	Z score	Syll	LWF	Typ	TDP	% Repeat	N-SOI	MI Total
Sub1	-2.50	0.05	0.05	0.05				3
Sub2	-2.48	0.05	0.05		0.05	0.05		2
Sub3	-2.17			0.05	0.05			0
Sub4	-2.11							0
Sub5	-2.11		0.05	0.05	0.05			1
Sub6	-1.99				0.05		*	0
Sub7	-1.94				0.05			-1
Sub8	-1.75			0.05	0.05	0.05		1
Sub9	-1.72				0.10	0.10		0
mal1	-3.02	0.05	0.05			0.05		3
mal2	-2.96	0.05	0.05	0.05		0.05		4
mal3	-2.53	0.05	0.05	0.05		0.05		4
mal4	-2.21	0.10	0.05	0.05		0.10		4
mal5	-2.17	0.05	0.05	0.05		0.10	*	5
mal6	-2.14		0.05	0.05	0.10	0.10		2
mal7	-2.13	0.05	0.05	0.05		0.10		4
mal8	-2.04		0.05					1
mal9	-2.00	0.05	0.05	0.05		0.10	*	5
mal10	-1.93	0.05	0.05	0.05		0.05		4
mal11	-1.92		0.05	0.05		0.05	*	4
mal12	-1.89	0.05	0.05	0.10		0.05	*	5
mal13	-1.89	0.05	0.05	0.10		0.05		4
mal14	-1.89			0.05	0.10		*	1
mal15	-1.69	0.05	0.05			0.05	*	4
mTBI	-2.23		0.05	0.05	0.10			1
sTBI	-2.47			0.05	0.05	0.05		1

Data are from subjects with abnormal z-scores including 9 control subjects, 15 subjects in simulated malingering conditions, and two patients with TBI. N-SOI: SOI within one standard deviation of the mean from Experiment 1. MI total = sum of individual signs of malingering. See Table 2 for other abbreviations.

We therefore investigated whether simulated malingerers with abnormal semantic z-scores could be distinguished from control subjects with abnormal z-scores based on the analysis of lexical, temporal, and ESA measures. Table 6 shows the subjects falling in the p<0.05 (shaded) and p<0.10 portions of the normative data distribution for measures of word syllable count, word frequency, word typicality, TDP, and the percentage of repeated words. It also shows subjects whose SOIs fell within the normal range (i.e., less than one standard deviation below the mean).

Simulated malingerers with abnormal scores used short, frequent, and typical words so that ancillary lexical measures showed moderate to good sensitivity and specificity in classifying subjects with abnormal scores into simulated malingering and control groups. For example, 73% of abnormal malingerers had mean syllable counts in the bottom 10% of the control distribution, a pattern that was seen in only 22% of the abnormal controls (i.e., 73% sensitivity and 78% specificity). Similarly, abnormally low word frequencies showed a sensitivity of 93% and a specificity of 67%, while typicality showed a sensitivity of 80% and specificity of 56%. An abnormally high percentage of repeated words provided 87% sensitivity and 67% specificity. In contrast, abnormally steep declines in the rate of word production (p< 0.10) were mainly seen in abnormal controls (78% sensitivity and 87% specificity), while SOIs within the normal range were more frequent among simulated malingerers (40% sensitivity and 89% specificity). Taking all six measures into account, malingering subjects with abnormal scores showed an average of 3.6 (sd = 1.3) signs of malingering, whereas abnormal controls showed only 0.7 (sd = 1.2) signs. A cutoff of three (of six) signs of malingering resulted in a sensitivity of 80% and a specificity of 89%.

### Experiment 3: Discussion

As in previous studies of simulated malingerers [96] and patients presumed to be malingering [97, 98], we found significant reductions in correct-word scores among simulated malingerers. However, z-score cutoffs were relatively ineffective at classifying subjects with abnormal performance into malingering and non-malingering groups. This insensitivity reflects the high coefficient of variation of correct-word scores in normative studies (see Table 1). In many neuropsychological tests, z-score cutoffs below -3.0 are needed to avoid falsely categorizing patients with abnormal performance as malingerers [75, 76]. However, to have a correct-word z-score of -3.0 in the current experiment, malingerers would need to retrieve fewer than 7.7 words in semantic conditions and only one word in phonemic conditions.

However, six other measures showed potential utility in distinguishing abnormal controls from abnormal malingerers, providing an aggregate sensitivity of 80% and a specificity of 89%. Virtually all malingerers adopted a lexical strategy: they used monosyllabic, frequent, and typical words which they often repeated. However, unlike abnormal control subjects, simulated malingerers with abnormal scores did not show abnormal declines in the rate of word production, and often had SOIs within the normal range. Thus, while malingerers produced abnormal correct-word scores, they did so in a manner that failed to match the characteristics of non-malingering subjects with abnormal scores. In other neuropsychological tests, ancillary performance measures have shown utility in distinguishing simulated malingerers and controls with abnormal scores [75, 78, 81, 99, 100]. Thus, while malingerers may produce criterion scores in the abnormal range they do so in different manner from subjects with intrinsically limited processing abilities.

#### Limitations

The subjects in Experiment 3 were familiar with C-VF test procedures, which may have influenced their performance and strategies. Further testing with naïve subjects in simulated malingering conditions and patients suspected of malingering is needed to validate these findings and determine if the proposed metrics provide similar sensitivity and specificity in identifying malingering subjects in different populations.

Although the malingering indices were effective in discriminating control subjects with abnormal performance from malingering subjects with abnormal performance, the false positive rate would be expected to increase significantly in more severely impaired clinical populations. For example, patients with AD retrieve relatively fewer items in the first half than the second half of the test [35]. Therefore, they would be expected to show reduced TDPs, similar to those of malingering subjects. Similarly, AD patients use more frequent and typical words [29] and show an increased incidence of repeated words [101].

### Experiment 4: The Effects of Traumatic Brain Injury

Verbal fluency tests are commonly used to assess executive and language functions in patients who have suffered traumatic brain injury (TBI). While patients with mild TBI (mTBI) show VF deficits in the acute phase [102], they typically perform within the normal range when tested more than six months post-injury [5, 103–105]. However, persistent deficits have been reported in subgroups of Veteran mTBI patients who fail to return to active duty [106], who have persistent memory problems [107], or who have suffered repeated blast exposure [108]. Reductions in the number of semantic switches [107] and semantic cluster size [46] have also been found in some studies. Verbal fluency deficits may also be more prominent in mTBI patients with a concurrent diagnosis of post-traumatic stress disorder (PTSD) [109–111].

Patients with severe TBI (sTBI) often show deficits in VF testing. In their meta-analysis, Henry and Crawford [5] found that patients with sTBI were comparably impaired on semantic and phonemic fluency tasks with an effect size (r = 0.46) similar to that seen in schizophrenia (r = 0.46) [15], but less than that seen in dementia (r = 0.55 for phonemic fluency and r = 0.72 for semantic fluency) [16], or following focal lesions of the left frontal or left temporal lobes (r = 0.58) [11]. More recent studies have also found reduced correct-word scores in sTBI patients [37, 112, 113], including one study that found greater reductions early in the test period [37]. Deficits increase in parallel with increasing TBI severity [46, 47], and include impairments in semantic organization [113].

### Experiment 4: Methods

#### Subjects and Procedures

The methods were identical to those used in Experiment 1. Twenty-five Veterans with a history of TBI were recruited from the Veterans Affairs Northern California Health Care System patient population. The patients included 24 males and one female between the ages of 20 and 61 years (mean age = 35.5 years), with an average education of 13.6 years. All patients had suffered one or more head injuries with a transient loss or alteration of consciousness, most related to blast exposure, and had received diagnoses after extensive clinical evaluations. All patients were tested at least one year post-injury. Twenty-one of the patients had suffered one or more combat-related incidents, with a loss of consciousness of less than 30 minutes, no hospitalization, and no evidence of brain lesions on clinical MRI scans. These patients were categorized as mTBI. The remaining four patients had suffered more severe accidents with hospitalization, coma duration exceeding eight hours, and post-traumatic amnesia exceeding 72 hours. These patients were categorized as sTBI. All patients were informed that the study was for research purposes only and that the results would not be included in their official medical records. Evidence of PTSD, as reflected in elevated scores (> 50) on the Posttraumatic Stress Disorder Checklist (PCL), was evident in the majority of the TBI sample (see S1 Table), producing highly significant differences in PCL scores between the TBI sample (mean 51.8, sd = 12.9) and the control subjects (mean = 32.0, sd = 12.8) in Experiment 1 [F(1,197) = 50.15, p< 0.0001, ω^2^ = 0.20] and Experiment 2a [F(1,62) = 57.48, p< 0.0001, ω^2^ = 0.47].

#### Statistical analysis

The results were analyzed with ANOVA, as in Experiment 1, with separate comparisons of mTBI and sTBI groups with the control subjects in Experiment 1 and Experiment 2a.

### Experiment 4: Results

Table 2 includes summary performance statistics for the mTBI and sTBI patients, and Fig 1 includes the correct-word scores (mTBI = solid red circles, sTBI = cross-hatched red circles). Fig 2 shows the correct-word z-scores of individual TBI patients in semantic and phonemic conditions. The majority of mTBI patients had correct-word z-scores within the normal range in both conditions (semantic mean = 0.20, phonemic mean = -0.15). The statistical analysis of correct-word z-scores with Group (mTBI, control) and Test-type (semantic, phonemic) as factors showed no significant overall differences between the mTBI patients and the control subjects in either Experiment 1 or Experiment 2a. Only one mTBI patient produced an abnormal (p<0.05) correct-word z-score in semantic testing without signs of malingering (see Table 6), while a separate mTBI patient showed abnormalities in phonemic testing.

Fig 3 shows the rate of word production in both conditions for mTBI patients (solid red lines): in both conditions, the decline in retrieval resembled that seen in control populations. In semantic conditions, the TDP was not significantly different from that of Experiment 1 subjects, but was marginally increased in comparison with subjects in Experiment 2a [F(1,42) = 4.49, p< 0.05, ω^2^ = 0.06].

Fig 4 shows the relationship between semantic z-scores and SOI scores. There were no significant differences between mTBI patients and the subjects in Experiment 1 or Experiment 2a in the SOI, word frequency, percentage of repeated words, or word syllable counts. The mTBI patients showed reduced word typicality in comparison with the subjects in Experiment 1 [F(1,199) = 29.37, p < 0.001, ω^2^ = 0.12], but no significant difference with the subjects in Experiment 2a. The number of semantic switches did not differ from those of Experiment 1 subjects, whether measured with ESA or Troyer methods, although the number of semantic switches was slightly reduced in comparison with the subjects in Experiment 2a [ESA: F(1,59) = 4.96, p < 0.03, ω^2^ = 0.06; Troyer: F(1,59) = 10.40, p < 0.005, ω^2^ = 0.14]. There were no changes in the number of ESA-defined multi-word clusters or cluster size in comparison with either Experiment 1 or Experiment 2a. Self-reported PTSD severity did not influence performance: We found no significant correlations between PCL scores and the correct-word scores for either mTBI patients or Experiment 1 controls in either the semantic or phonemic tests.

Unlike mTBI patients, sTBI patients showed significant correct-word z-score reductions (semantic = -1.01, phonemic = -0.74) with respect to the control subjects in Experiment 1 [F(1,182) = 4.52, p<0.05, ω^2^ = 0.02] and Experiment 2a [F(1,42) = 6.08, p < 0.02, ω^2^ = 0.11], without significant Group x Test-type interactions. Of the four sTBI patients, one produced significant abnormalities on semantic testing and borderline (p < 0.07) abnormalities in phonemic testing, two others showed smaller performance impairments on both tests, and one patient showed average performance.

The number of switches during semantic testing was reduced in sTBI patients compared to the subjects in Experiment 1 [ESA: F(1,182) = 6.33, p<0.02, ω^2^ = 0.03; Troyer F(1,182) = 3.42, p<0.07] and Experiment 2a [ESA: F(1,42) = 10.50, p < 0.005, ω^2^ = 0.18; Troyer: F(1,42) = 7.30, p < 0.02, ω^2^ = 0.13]. The number of ESA-defined multi-word clusters did not differ from Experiment 1, but was reduced with respect to Experiment 2a [F(1,42) = 4.85, p < 0.05, ω^2^ = 0.08]. Typicality showed a similar pattern [vs. Experiment 1 subjects, F(1,182) = 1.68, NS; vs. Experiment 2a subjects, F(1,42) = 5.90, p < 0.03, ω^2^ = 0.10]. The abnormalities in sTBI patients occurred without significant alterations in the size of ESA-defined multi-word clusters in comparison with either control group. No group differences were seen in the SOI, word frequency, word syllable count, percentage of repeated words, or the TDP. The TBI patients with abnormal z-scores in semantic testing did not show signs of malingering (see Table 6).

### Experiment 4: Discussion

We found no systematic group differences in correct-word z-scores in military veterans with mTBI when compared to the control subjects of Experiment 1 or Experiment 2a. Nor did the mTBI patients show consistent alterations in the number of semantic switches, the number of multi-word clusters, multi-word cluster size, the SOI, the percentage of repeated words, word frequency, or word length. This is consistent with the results of the large scale study of Vanderploeg et al. (2005) [104], who found similar VF performance in 254 Veteran patients with mTBI and 3,057 control veterans. Like many of the Veteran patients tested by Vanderploeg et al. (2005), a high percentage of Veteran patients in Experiment 4 had co-morbid PTSD and elevated PCL scores. However, we found no evidence that elevated PCL scores reduced correct-word scores in either the control subjects or the mTBI patients.

In contrast, the sTBI patients showed deficits in both semantic and phonemic tests. In semantic testing, the number of switches was significantly reduced, with abnormalities seen in 75% of the sTBI group. Similar deficits in semantic switching have been reported in previous studies of patients with moderate and severe TBI [46, 47, 113]. The pattern of results is similar to those observed in patients with frontal lobe lesions [40], and is consistent with quantitative neuroimaging studies that revealed extensive frontal lobe damage in the most impaired sTBI patient in Experiment 4 [114]. We also found corresponding decreases in the number, but not the size, of multi-word clusters, and increases in word typicality without significant alterations in the SOI, the percentage of repeated words, word frequency, word length, or the TDP.

#### Limitations

These results should be considered preliminary, given the small sample size of the TBI patient populations. Additional studies will be needed to investigate the sensitivity of different C-VF measures in other clinical populations.

## General Discussion

Verbal fluency tests are among the fastest and easiest neuropsychological tests to administer and score. Testing usually requires 60 to 90 s per category, and tallying the number of correct words and repetitions can be performed rapidly. Evaluating correct-word scores relative to tabulated data is also straightforward, although test interpretation may differ somewhat depending on the normative data used for comparison.

The C-VF is as easy to administer than paper-and-pencil VF tests, and offers several additional improvements: (1) A permanent record of test performance is stored digitally; (2) Timing is recorded automatically so that words can be accurately assigned to 15 second intervals; (3) Scoring of correct and repeated words is performed automatically; and (4) Z-scores based on an individual’s age, education, and computer-use are produced that are somewhat more precise than comparisons with tabulated correct-word scores based on subjects spanning a range of ages and educational levels.

However, the main advantage of the C-VF is the comprehensive set of lexical, temporal, and semantic measures that it provides. These measures include word frequency, syllable count, typicality, and the TDP. In addition, the application of Explicit Semantic Analysis [57] makes it possible to objectively analyze the semantic relationships between words in any semantic category, quantify semantic organization with the SOI, and measure semantic switches, multi-word clusters, and multi-word cluster size. The C-VF Python program also performs switch and cluster analysis in the “animals” category using predefined Troyer semantic subcategories [39].

From the perspective of the subject, the only difference between the C-VF and a standard VF assessment is test duration (90 vs 60 seconds). Correct-word scores over the first 60 s of the C-VF were similar to the average scores obtained in large-scale VF studies. We also found similar demographic correlates: age and sex did not have significant influences on correct-word scores, while education showed a correlation similar to that observed in previous studies. An additional factor, daily computer-use, also correlated significantly with performance.

Correct-word z-scores, created after factoring out the influence of education and computer-use on performance, generalized across control populations in Experiment 1 and Experiment 2a. The test-retest reliability of the C-VF correct-word scores equaled or exceeded that of manually scored VF tests. Repeat testing in Experiment 2 using different semantic and phonemic categories showed high intraclass correlation coefficients for measures of word frequency, syllable count, and typicality.

### ESA measures of semantic organization

The semantic organization index (SOI), the ratio of semantic association strength between successive words (PW-ESA) relative to the average association strength among all words (A-ESA), was strongly associated with correct-word scores. Subjects with greater correct-word scores produced clusters of highly related words (high PW-ESA) and, above all, produced words in more semantically distinct clusters (low A-ESA).

The SOI is an appealing measure of semantic organization because it less computationally confounded with correct-word scores than measures of semantic switches and multi-word clusters; e.g., a high SOI can occur with low correct-word scores, but high semantic switch and multiword cluster scores will be obligatorily associated with high correct-word scores. The SOI showed good test-retest reliability across different word lists in Experiment 2, suggesting that it is a stable characteristic of individual subjects Finally, in phonemic fluency tests, the SOI revealed that the semantic relationships between words influenced the order of word recall, consistent with previous studies showing semantic influences during phonemic fluency conditions [26].

### Measures of semantic switches and clusters

In semantic tests of the “animals” category, ESA measures of semantic switches and clusters correlated strongly with corresponding measures obtained with the method of Troyer et al. (1997) [39]. However, unlike subcategory-based methods, ESA measures of switches and clusters can be automatically analyzed for novel categories. In Experiment 2, we were able to establish that the number of ESA-defined switches and clusters showed significant correlations across different semantic categories including two that lacked pre-defined semantic subcategories. Moreover, ESA can be applied to categories of different size. For example, ESA will reveal the semantic organization of report in subjects who names animals from a single Troyer subcategory (e.g., “North American Animals”)

### Clinical applications

The utility of the C-VF performance measures was shown in Experiment 3, where 38% of simulated malingerers showed abnormal correct-word z-scores. Simulated malingerers with abnormal scores were not well-distinguished from control subjects with abnormal scores based on correct-word z-score cutoffs alone. However, they could be distinguished with 80% sensitivity and 89% specificity based on a combination of other measures including word frequency, syllable count, typicality, the TDP, semantic organization, and the percentage of repeated words.

In Experiment 4, patients with mild TBI performed within the normal range on almost all measures. However, patients with sTBI showed significant abnormalities in correct-word z-scores and significant reductions in the number of semantic switches. Further investigation is needed to evaluate C-VF measures in clinical disorders such as mild cognitive impairment, Alzheimer’s disease, and schizophrenia.

### Future directions

We have adapted the C-VF to a Microsoft Surface Pro to enhance portability and ease of administration. We have also added optional digital recording of the subject’s spoken responses and voice trigger detection to improve response-timing measures. We plan to gather additional control data and are looking forward to assisting interested investigators in evaluating C-VF sensitivity in different clinical and control populations.

## Conclusion

Computerized transcription and analysis of responses during verbal fluency testing facilitates test administration, speeds scoring, and provides additional objective and reliable measures of lexical, temporal, and semantic processing in normal subjects, simulated malingerers, and patients with traumatic brain injury.

## Supporting Information

S1 Fig Correct word scores in semantic (“animals”) and phonemic (letter “F”) conditions as a function of years of education. The data are from Experiment 1, Experiment 2a, Experiment 3 (simulated malingering) and Experiment 4 (mild TBI = mTBI, filled red circles; severe TBI = sTBI, cross-hatched red circles). The regression slope is from Experiment 1.
(TIF)

S2 Fig Correct word scores in semantic (“animals”) and phonemic (letter “F”) conditions as a function of daily hours of computer-use. The data are from Experiment 1, Experiment 2a, Experiment 3 (simulated malingering) and Experiment 4 (mild TBI = mTBI, filled red circles; severe TBI = sTBI, cross-hatched red circles). The regression slope is from Experiment 1.
(TIF)

S1 Table PATIENT CHARACTERISTICS. Shaded cells show patients with severe TBI. Edu = years of education.C-use: computer-use. PCL = scores on post-traumatic stress disorder checklist. CW-S = correct words in semantic test;CW-P = correct words in phonetic test.
(DOCX)
