# Supplementary figures and images for: Computerized Analysis of Verbal Fluency: Normative Data and the Effects of Repeated Testing, Simulated Malingering, and Traumatic Brain Injury
Source: PLoS One. 2016 Dec 9;11(12):e0166439. doi: 10.1371/journal.pone.0166439 (PMC5147824; doi:10.1371/journal.pone.0166439)

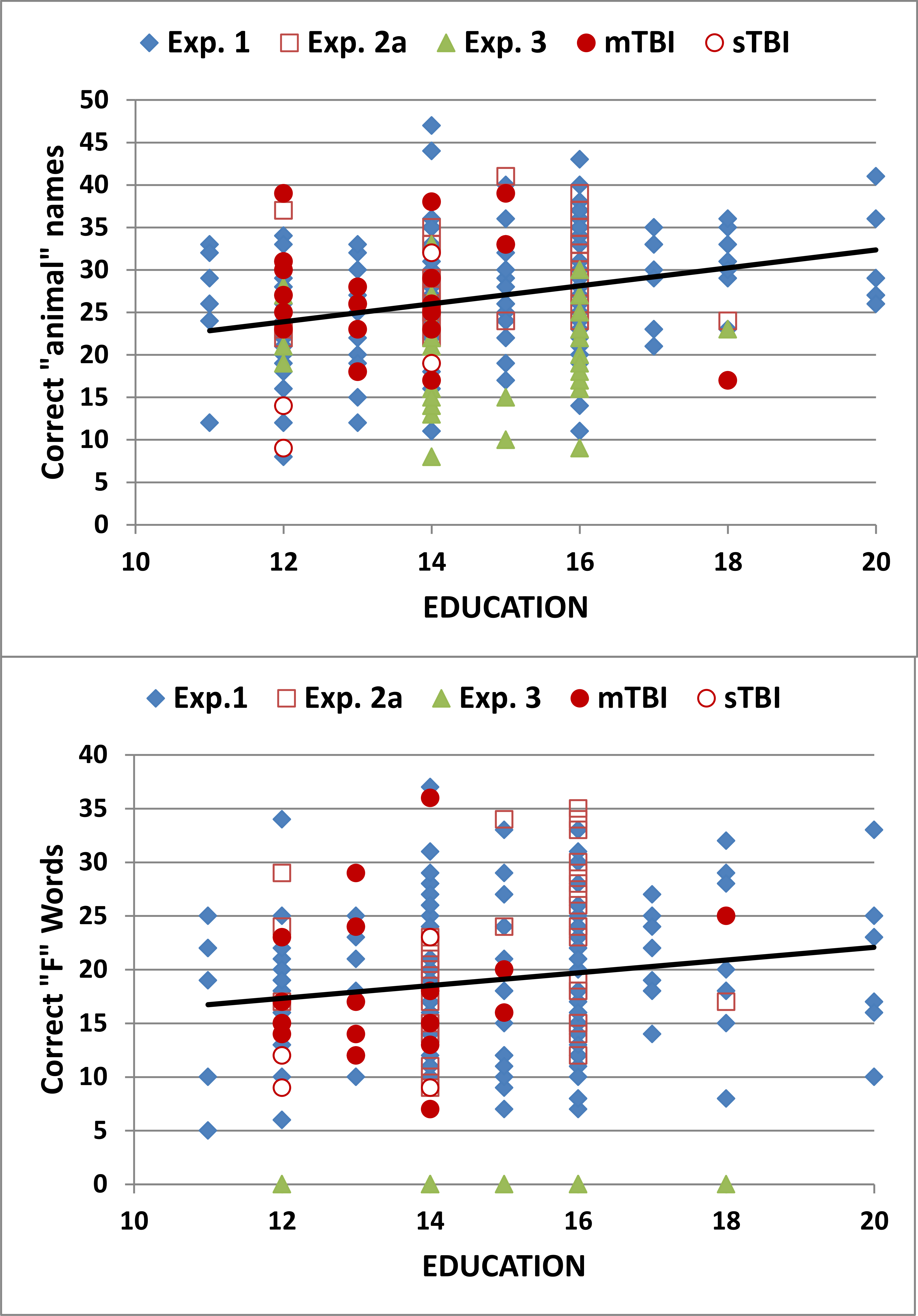

Supplement: S1 Fig — The data are from Experiment 1, Experiment 2a, Experiment 3 (simulated malingering) and Experiment 4 (mild TBI = mTBI, filled red circles; severe TBI = sTBI, cross-hatched red circles). The regression slope is from Experiment 1. (TIF) [file pone.0166439.s001.tif]

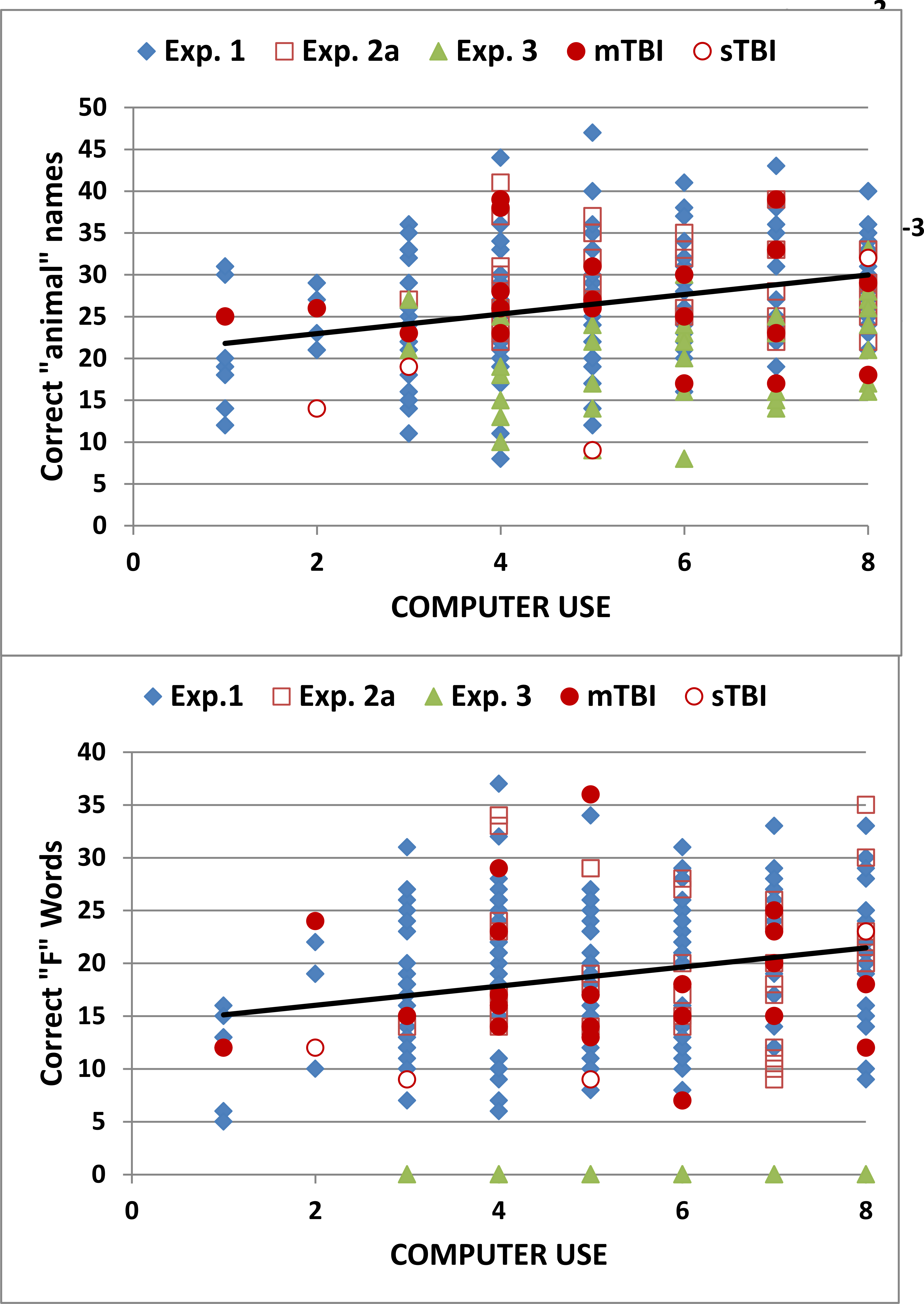

Supplement: S2 Fig — The data are from Experiment 1, Experiment 2a, Experiment 3 (simulated malingering) and Experiment 4 (mild TBI = mTBI, filled red circles; severe TBI = sTBI, cross-hatched red circles). The regression slope is from Experiment 1. (TIF) [file pone.0166439.s002.tif]
