# Supplementary material for: Computerized Analysis of Verbal Fluency: Normative Data and the Effects of Repeated Testing, Simulated Malingering, and Traumatic Brain Injury
Source: PLoS One. 2016 Dec 9;11(12):e0166439. doi: 10.1371/journal.pone.0166439 (PMC5147824; doi:10.1371/journal.pone.0166439)
Supplement: S1 Table — Shaded cells show patients with severe TBI. Edu = years of education.C-use: computer-use. PCL = scores on post-traumatic stress disorder checklist. CW-S = correct words in semantic test;CW-P = correct words in phonetic test. (DOCX) [file pone.0166439.s003.docx]

|  | Age | Edu | C-use | PCL | CW-S | CW-P |
| --- | --- | --- | --- | --- | --- | --- |
| PAT1 | 24 | 12 | 5 | 54 | 27 | 14 |
| PAT2 | 31 | 13 | 4 | 28 | 26 | 14 |
| PAT3 | 52 | 14 | 7 | 27 | 23 | 15 |
| PAT4 | 25 | 15 | 7 | 0 | 33 | 20 |
| PAT5 | 28 | 13 | 4 | 47 | 23 | 17 |
| PAT6 | 25 | 12 | 6 | 57 | 30 | 15 |
| PAT7 | 29 | 12 | 7 | 54 | 39 | 23 |
| PAT8 | 47 | 14 | 5 | 52 | 26 | 13 |
| PAT9 | 28 | 14 | 5 | 43 | 26 | 36 |
| PAT10 | 29 | 13 | 4 | 27 | 28 | 29 |
| PAT11 | 61 | 18 | 7 | 52 | 17 | 25 |
| PAT12 | 27 | 15 | 4 | 72 | 39 | 16 |
| PAT13 | 48 | 13 | 8 | 59 | 18 | 12 |
| PAT14 | 49 | 12 | 1 | 47 | 25 | 12 |
| PAT15 | 28 | 14 | 4 | 68 | 38 | 23 |
| PAT16 | 39 | 13 | 2 | 64 | 26 | 24 |
| PAT17 | 25 | 12 | 3 | 72 | 23 | 15 |
| PAT18 | 45 | 14 | 8 | 60 | 29 | 18 |
| PAT19 | 23 | 14 | 6 | 67 | 17 | 7 |
| PAT20 | 29 | 14 | 6 | 41 | 25 | 18 |
| PAT21 | 28 | 12 | 5 | 46 | 31 | 17 |
| PAT22 | 35 | 12 | 5 | 59 | 9 | 9 |
| PAT23 | 46 | 12 | 2 | 42 | 14 | 12 |
| PAT24 | 57 | 14 | 3 | 56 | 19 | 9 |
| PAT25 | 46 | 14 | 8 | 50 | 32 | 23 |
